# Molecular engineering and sequential cosensitization for preventing the “trade-off” effect with photovoltaic enhancement†
†Electronic supplementary information (ESI) available: Synthesis and characterization, and additional photovoltaic data. See DOI: 10.1039/c6sc03938c



**DOI:** 10.1039/c6sc03938c

**Published:** 2016-11-17

**Authors:** Weiwei Zhang, Yongzhen Wu, Xin Li, Erpeng Li, Xiongrong Song, Huiyun Jiang, Chao Shen, Hao Zhang, He Tian, Wei-Hong Zhu

**Affiliations:** a Shanghai Key Laboratory of Functional Materials Chemistry , Key Laboratory for Advanced Materials and Institute of Fine Chemicals , Collaborative Innovation Center for Coal Based Energy (i-CCE) , School of Chemistry and Molecular Engineering , East China University of Science and Technology , Shanghai 200237 , P. R. China . Email: whzhu@ecust.edu.cn; b Division of Theoretical Chemistry and Biology , School of Biotechnology , KTH Royal Institute of Technology , SE-10691 Stockholm , Sweden

## Abstract

In dye-sensitized solar cells (DSSCs), it is essential to use rational molecular design to obtain promising photosensitizers with well-matched energy levels and narrow optical band gaps. However, the “trade-off” effect between the photocurrent and photovoltage is still a challenge. Here we report four benzoxidazole based D–A–π–A metal-free organic dyes (**WS-66**, **WS-67**, **WS-68** and **WS-69**) with different combinations of π-spacer units and anchoring-acceptor groups. Either extending the π-spacer or enhancing the electron acceptor can efficiently modulate the molecular energy levels, leading to a red-shift in the absorption spectra. The optimal dye, **WS-69**, containing a cyclopentadithiophene (CPDT) spacer and cyanoacetic acid acceptor, shows the narrowest energy band gap, which displays a very high photocurrent density of 19.39 mA cm^–2^, but suffers from a relatively low photovoltage of 696 mV, along with the so-called deleterious “trade-off” effect. A cosensitization strategy is further adopted for enhancing the device performance. Optimization of the dye loading sequence is found to be capable of simultaneously improving the photocurrent and photovoltage, and distinctly preventing the “trade-off” effect. The superior cosensitized cell exhibits an excellent power-conversion efficiency (PCE) of 10.09% under one-sun irradiation, and 11.12% under 0.3 sun irradiation, which constitutes a great achievement in that the efficiency of a pure metal-free organic dye with iodine electrolyte can exceed 11% even under relatively weak light irradiation. In contrast with the previous cosensitization strategy which mostly focused on compensation of light-harvesting, we propose a novel cosensitization architecture, in which the large molecular-sized, high photocurrent dye **WS-69** takes charge of broadening the light-harvesting region to generate a high short-circuit current (*J*_SC_) while the small molecular-sized, high photovoltage dye **WS-5** is responsible for retarding charge recombination to generate a high open-circuit voltage (*V*_OC_). In addition, adsorption amount and photo-stability studies suggest that the cyano group in the anchoring acceptor is important for the stability since it is beneficial towards decreasing the LUMO levels and enhancing the binding of dyes onto TiO_2_ nanocrystals.

## Abstract 2

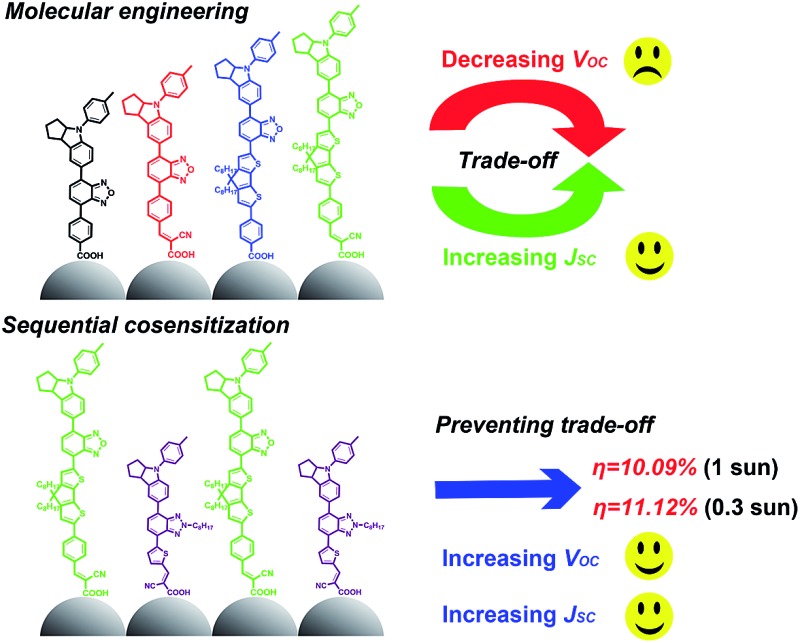
Cosensitization of two dyes with different molecular sizes and photovoltaic characteristics is demonstrated to successfully prevent the “trade-off” effect, leading to an excellent power-conversion efficiency of 10.09% under one-sun and 11.12% under 0.3 sun 4 irradiation.

## Introduction

Dye-sensitized solar cells (DSSCs) have attracted a large amount of attention due to their high potential for converting sunlight to electricity, easy fabrication and good stability.^1^ As one of the pivotal components in DSSCs, photosensitizers have been widely exploited and achieved considerably high power-conversion efficiencies (PCEs).^2^ To date, metal-based sensitizers, such as ruthenium polypyridine or zinc porphyrin, have achieved relatively high PCEs of 11–13%.^3^ However, due to the environmental impact and cost issues associated with these sensitizers, increased attention has been paid to pure metal-free organic sensitizers, which possess merits of being made from abundantly available raw materials, being environmentally benign, and having tunable molecular structures and optical properties.^4^ Nevertheless, metal-free organic dyes have scarcely achieved comparably high efficiency as metal-based sensitizers in DSSCs, especially in the case of the iodine electrolyte.^5^

As the key factor that determines the PCE, the photocurrent density depends largely on the light-harvesting abilities of photosensitizers as well as their highest occupied molecular orbital (HOMO) and lowest unoccupied molecular orbital (LUMO) energy levels.^6^ Generally, broad absorption spectra can be obtained through decreasing the band gap between the HOMO and LUMO orbitals of the photosensitizers.^7^ Meanwhile, a potential offset is required to act as the driving force for electron injection from the excited dyes to the TiO_2_ conduction band (0.5 V *vs.* NHE) and dye regeneration by accepting electrons from an iodine electrolyte (0.4 V *vs.* NHE). The mismatched LUMO and HOMO can always lead to inferior photocurrent performance due to the problematic electron injection or dye regeneration process.^8^ Therefore, it is particularly essential to use rational molecular design to obtain superior photosensitizers with well-matched energy levels and narrow optical band gaps.

Simultaneously, when extending the absorption spectrum of a sensitizer to obtain a higher photocurrent, a significant photovoltage loss is usually observed due to the prolific “trade-off” effect between the photocurrent and photovoltage. For example, Hua *et al.* ^9^ designed three quinoxaline-based organic dyes, **AQ201**, **AQ202**, and **AQ203**, by introducing different π-bridges to extend the conjugations. The gradually improved photocurrent from **AQ201** to **AQ203** is accompanied with a reduced photovoltage by 40–70 mV, eventually leading to a decreased PCE for **AQ203**. Similarly, for porphyrin sensitizers, Diau *et al.* ^10^ extended the π-conjugation of **LD-14** by coupling two zinc porphyrin cores *via* an acetylene bridge, bringing forth an improved photocurrent, but also a decrease in both the photovoltage and PCE. Even for the leading porphyrin dye **SM315**,^11^ which contains an electron-withdrawing unit of benzothiadiazole to broaden the light-harvesting ability, displays an enhanced photocurrent but reduced photovoltage, albeit achieving a high efficiency of 13%. Further, for squaraine dye, Han *et al.* ^12^ developed a series of near-infrared (NIR) squaraine sensitizers by incorporating different electron-deficient units. **HSQ4** with the broadest absorption and highest photocurrent density shows the weakest photovoltage due to faster charge recombination. Obviously, due to the “trade-off” effect, most low energy gap sensitizers often enhance the photocurrent at the cost of the photovoltage.^13^ Although the increase in the photocurrent sometimes overweighs the decrease in photovoltage, leading to a slightly improved PCE, there is no doubt that the “trade-off” effect can still be considered as the great and eternal barrier for further improving the PCE of DSSCs as well as for the future development of solar cells.

**Fig. 1:**
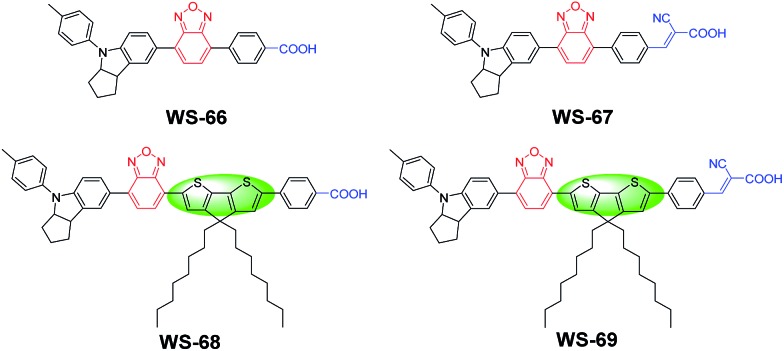
Molecular structures of dyes **WS-66**, **WS-67**, **WS-68** and **WS-69** with different combinations of π-spacer units and anchoring-acceptor groups.

In this work, we first optimize the energy levels of benzoxidazole (BOD) based D–A–π–A organic sensitizers by combining different π-spacer units and anchoring-acceptor groups. To illustrate their influence on molecular energy levels and absorption spectra, we design a simple dye **WS-66** (Fig. 1) as a reference. Replacing the carboxylic acid group in **WS-66** with a stronger anchoring group of cyanoacetic acid leads to the dye **WS-67**, which shows extended absorption spectra as a result of the downwards-shifted LUMO level. On the other hand, introducing an electron-rich unit of 4*H*-cyclopenta[2,1-*b*:3,4-*b*′]dithiophene (CPDT) into the π-spacer can lead to an upwards shift of the HOMO levels and further red-shift the absorption spectra, such as in dyes **WS-68** and **WS-69**. Preliminary device studies indicate that **WS-69** is the optimal of the four dyes, which shows a PCE of 9.03% with a high short-circuit current (*J*_SC_) of 19.39 mA cm^–2^ due to its broad spectral response. However, suffering from the “trade-off” effect, devices based on **WS-69** show a decreased open-circuit voltage (*V*_OC_) compared with the reference dye **WS-66**. To further enhance the device performance, we employ a co-sensitization strategy and carefully optimize the dye loading sequence. As demonstrated, a suitable dye loading sequence can simultaneously improve the *J*_SC_ and *V*_OC_, significantly overcoming the known “trade-off” effect between the two parameters. As a result, we achieved an excellent PCE of 10.09% under full AM 1.5G irradiation, and 11.12% under 0.3 sunlight. To the best of our knowledge, this is the first time that the efficiency of a pure metal-free organic dye with an iodine electrolyte exceeds 11% even under relatively weak light irradiation. Moreover, the adsorption amount and photo-stability studies suggest that the cyano group in the anchoring acceptor is important for the stability since it is beneficial to decreasing the LUMO levels and enhancing the binding of dyes onto the TiO_2_ nanocrystals.

## Results and discussion

### Molecular engineering with different combinations of π-spacer units and anchoring-acceptor groups

D–A–π–A-based organic dyes have been widely developed due to their ability to facilitate electron migration in excited dye as well as their higher stability with respect to traditional D–π–A sensitizers.^14^ In this regard, compared to the commonly used benzothiadiazole (BTD) auxiliary acceptor,^15^ the stronger benzoxidiazole (BOD) unit remains relatively unexplored. Hence, in this context, the BOD additional acceptor is chosen to bridge with the powerful donor of an indoline unit, in combination with a different anchoring group and π-bridge. As is well known, carboxylic acids are widely used in ruthenium polypyridine or zinc porphyrin dyes,^16^ while cyanoacetic acid is commonly used in metal-free organic dyes.^17^ To investigate dye characteristics brought about by different anchoring units, four sensitizers were designed with these two different anchoring groups. Moreover, based on **WS-66** and **WS-67** with a benzene spacer, an efficient building block octyl-decorated CPDT unit was further introduced into **WS-68** and **WS-69** to enhance their electron-donating ability. The synthetic routes of the four sensitizers are outlined in Scheme 1 in the ESI†. The target sensitizers **WS-66** and **WS-67** started from 4-bromo-7-(4-(*p*-tolyl)-1,2,3,3*a*,4,8*b*-hexahydrocyclopenta[*b*]indol-7-yl)benzo[*c*][1,2,5]oxadiazole (**1**), which is a quite stable red powder and does not undergo colour change for at least one year, mainly owing to the strong electron-withdrawing BOD unit.^18^ It is first converted to compounds **3** and **5** by Suzuki coupling with butyl 4-(4,4,5,5-tetramethyl-1,3,2-dioxaborolan-2-yl)benzoate (**2**) and (4-formylphenyl)boronic acid (**4**), respectively. Afterwards, compound **3** was converted to sensitizer **WS-66** by ester hydrolysis in KOH with a high yield of 75.9%, while compound **5** was converted to sensitizer **WS-67** by Knoevenagel condensation with cyanoacetic acid with a yield of 68.8%. Compound **8** (purple powder) was obtained from compound **1** and CPDT (**6**) *via* a Suzuki reaction and then bromination. The bromination reaction was conducted without purification for a high conversion yield. Then **8** was converted to **9** and **10** by Suzuki coupling with **2** and **4**, which is similar to the synthesis of **3** and **5**. Likewise, the precursors, **9** and **10**, were converted to **WS-68** and **WS-69** by ester hydrolysis and Knoevenagel condensation, respectively. All intermediates and the target sensitizer dyes were characterized by ^1^H and ^13^C NMR, and high-resolution mass spectroscopy (HR-MS). Their corresponding spectra are presented in the ESI.†

### Enhanced light-harvesting from a narrowed energy band gap

**Fig. 2:**
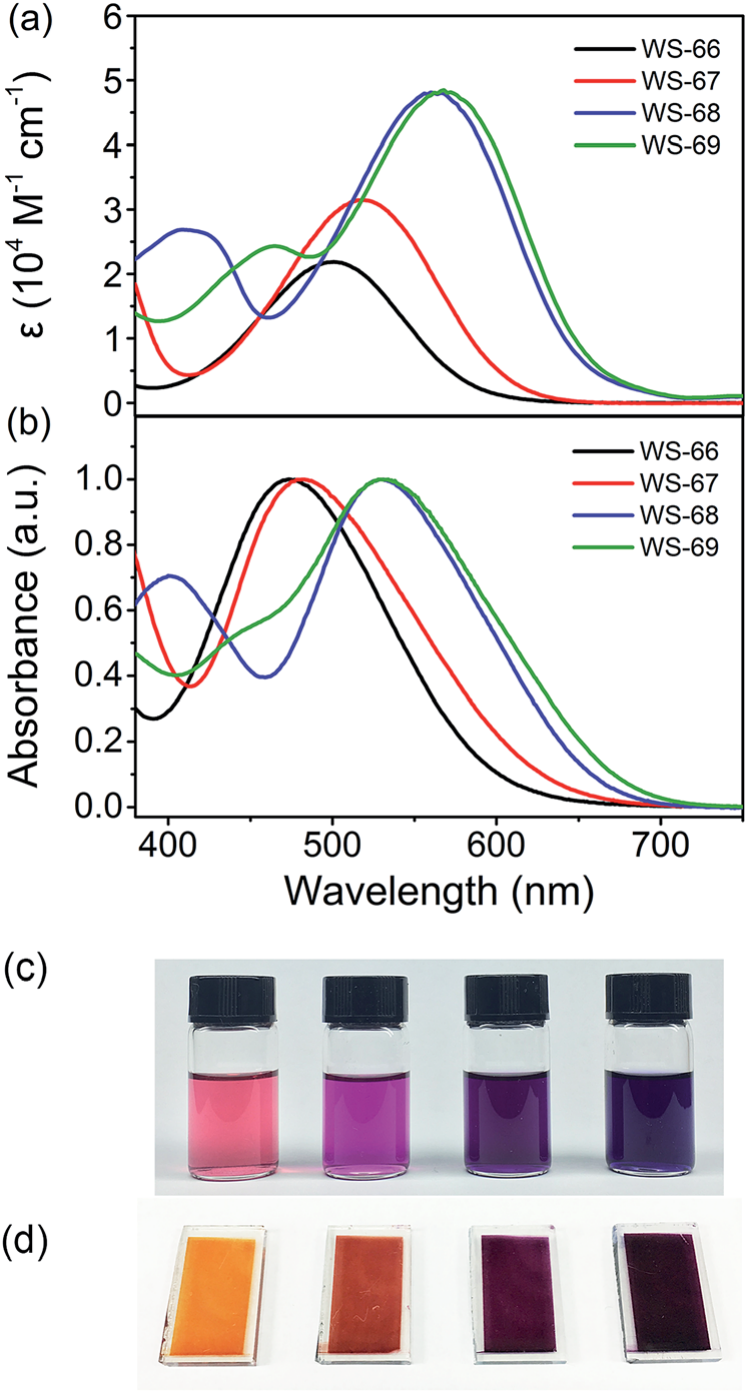
Absorption spectra of dyes **WS-66**, **WS-67**, **WS-68** and **WS-69** in CH_2_Cl_2_ (a) and on 3 μm TiO_2_ films (b). Photographs of dyes **WS-66**, **WS-67**, **WS-68** and **WS-69** (from left to right) in solution (c) and on TiO_2_ films (d).

Fig. 2 shows the UV-Vis absorption spectra of dyes **WS-66**, **WS-67**, **WS-68** and **WS-69** measured in CH_2_Cl_2_ solution (3 × 10^–5^ M, Fig. 2a) and on TiO_2_ films (3 μm, Fig. 2b). Their corresponding characteristic data are summarized in Table 1. **WS-66** shows a distinct absorption band from 380 to 650 nm, with an intramolecular charge transfer (ICT) peak located at 501 nm. When the carboxylic acid group in **WS-66** is replaced by the much stronger electron acceptor of cyanoacetic acid, as in **WS-67**, it displays a bathochromic shift of 18 nm in the ICT peak, with a maximum absorption band at 519 nm. Dye **WS-68**, with an inserted electron-rich group of CPDT in the π-spacer, exhibits a more obvious red-shift by 59 nm in the ICT peak (up to 560 nm), along with an enhanced molar extinction coefficient (*ε*, 4.81 × 10^4^ M^–1^ cm^–1^). The insertion of the CPDT unit is expected to relieve the steric hindrance between BOD and the benzene unit and to enhance the conjugation, thus facilitating the ICT process. Further replacing the carboxylic acid group in **WS-68** with cyanoacetic acid to give dye **WS-69** leads to a slight bathochromic shift of 8 nm in the ICT peak to 568 nm. Overall, it can be found that the ICT band is gradually red-shifted with an improved absorption coefficient from **WS-66** to **WS-69**, which can be reflected in a photograph of the dye solution, changing from pink to purple then to dark violet (Fig. 2c). These results indicate that the introduction of a CPDT unit into the D–A–π–A framework combined with a cyanoacetic acid acceptor is beneficial to broadening the absorption and enhancing the light-harvesting capability.

**Table 1:** Optical and electrochemical data for dyes **WS-66**, **WS-67**, **WS-68** and **WS-69**

Dye	*λ*_max_ ^*a*^ [nm]	*ε* ^*a*^ [M^–1^ cm^–1^]	*λ*_max_ on TiO_2_ ^*b*^ [nm]	HOMO^*c*^ [V] (*vs.* NHE)	*E*_0–0_ ^*d*^ [eV]	LUMO^*e*^ [V] (*vs.* NHE)
**WS-66**	501	21 898	473	0.99	2.07	–1.08
**WS-67**	519	31 495	483	0.99	1.98	–0.99
**WS-68**	560	48 122	530	0.87	1.89	–1.02
**WS-69**	568	48 465	532	0.87	1.85	–0.98

^*a*^Absorption peaks (*λ*_max_) and molar extinction coefficients (*ε*) were obtained in CH_2_Cl_2_.

^*b*^Absorption parameters were obtained on 3 μm TiO_2_ films.

^*c*^The HOMO was obtained in CH_2_Cl_2_ (working electrode: glassy carbon; reference electrode: saturated calomel electrode (SCE); counter electrode: Pt); the measured redox potentials were calibrated with ferrocene as an external reference and converted to NHE by addition of +0.69 V.

^*d*^ *E*_0–0_ values were estimated from the wavelength at 10% maximum absorption intensity for the dye-adsorbed 3 μm TiO_2_ film.

^*e*^The LUMO was calculated according to LUMO = HOMO – *E*_0–0_.

Upon adsorption onto TiO_2_ films, all four dyes show hypsochromic shifts in their ICT bands, which can be ascribed to deprotonation of the carboxylic acid or cyanoacetic acid. Meanwhile, there is a variation tendency similar to that occurring in solution with a gradual bathochromic shift in the ICT peak can be observed from **WS-66** (473 nm) to **WS-69** (532 nm). As is shown in Fig. 2d, the colour of the dye-loaded TiO_2_ films changes from orange to red then to purple gradually. The light-harvesting efficiency (LHE, Fig. S1†) calculated from Fig. 2b well matches the behaviour of the absorption spectra, reaching close to 100% near the ICT band even for the 8 μm TiO_2_ film. Practical light-harvesting in solar cells should be more efficient due to the thicker mesoscopic TiO_2_ film (16 μm) used in devices.

### Modulation of energy levels from the LUMO to HOMO

**Fig. 3:**
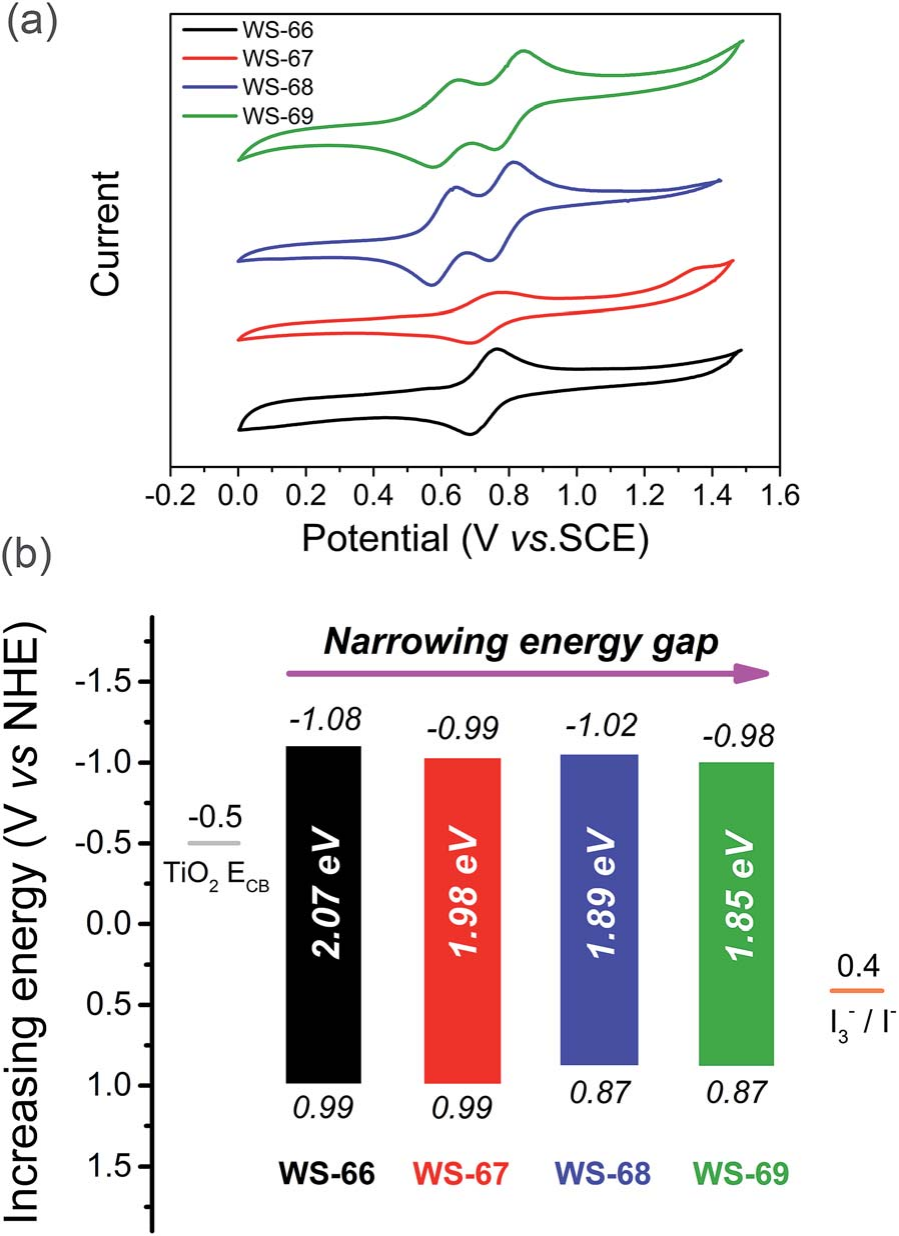
Cyclic voltammograms of dyes **WS-66**, **WS-67**, **WS-68** and **WS-69** measured in CH_2_Cl_2_ (a), LUMO (above the coloured bar) and HOMO (under the coloured bar) values, and energy band gaps of dyes **WS-66**, **WS-67**, **WS-68** and **WS-69** (b).

Generally, the energy gap between the LUMO and HOMO is calculated from the absorption edge of dye-loaded TiO_2_ film.^19^ The broader the absorption the sensitizer exhibited, the narrower the energy gap it actually possesses. Meanwhile, the LUMO and HOMO levels of the sensitizers that critically determine photo-generated electron-injection and dye-regeneration play a pivotal role in the ultimate power output of the devices. Accordingly, there is a persistent pursuit to design photosensitizers with a well-matched energy level and narrower band gap *via* molecular engineering. Here, to thermodynamically evaluate dye regeneration and charge injection, the electrochemical properties of four dyes, **WS-66**, **WS-67**, **WS-68** and **WS-69**, were measured by cyclic voltammetry in CH_2_Cl_2_ with tetra-*n*-butylammonium hexafluorophosphate (0.1 M, Fig. 3a). The SCE reference electrode was calibrated using a ferrocene redox couple as an external standard and the *E*_1/2_ of the ferrocene redox couple was found to be 0.42 V *versus* the SCE reference electrode. The potentials *versus* the NHE were calibrated by the addition of 0.69 V to the potentials *versus* ferrocene. The energy level data (LUMO, HOMO and energy band gap) are listed in Table 1 and Fig. 3b. The LUMO and HOMO values for reference dye **WS-66** are –1.08 and 0.99 V, respectively, along with a wide energy gap of 2.07 eV. It is generally considered that the energy values for the TiO_2_ conduction band and iodine electrolyte in DSSCs are –0.5 V and 0.4 V, respectively. And the minimum driving force requirement for excited electron injection into TiO_2_ and dye regeneration by the electrolyte is about 0.3 V.^8a,20^ Therefore, **WS-66** theoretically possesses sufficient LUMO and HOMO values for electron-injection and dye-regeneration with a driving force of more than 0.5 V. Based on **WS-66**, replacing carboxylic acid with a stronger electron-withdrawing acceptor of cyanoacetic acid induces a downwards shift of the LUMO level by 0.09 V, while no variation in the HOMO level was observed, resulting in a reduced band gap (1.98 eV) for **WS-67**. Considering the relatively positive HOMO levels for **WS-66** and **WS-67** which have the potential to be further modulated, the electron-rich CPDT unit was purposefully introduced into the π-bridge to upwards shift the HOMO level. As we expected, the HOMO levels for **WS-68** and **WS-69** are successfully uplifted by 0.12 V with respect to **WS-66** and **WS-67**. In particular, the optimal dye, **WS-69**, displays the narrowest band gap (1.85 eV) of the four sensitizers, along with theoretically enough driving force for electron-injection (0.48 V) and dye-regeneration (0.47 V). Comparing these four dyes, the customized LUMO levels downwards shift from –1.08 V (**WS-66**) to –0.98 V (**WS-69**), and the customized HOMO levels upwards shift from 0.99 V (**WS-66**) to 0.87 (**WS-69**), together with the energy gap reducing from 2.07 eV (**WS-66**) to 1.98 eV (**WS-67**) to 1.89 eV (**WS-68**) to 1.85 eV (**WS-69**). Here, for the metal-free organic dye, we have successfully demonstrated the rational modulation of energy level for a narrower band gap dye through molecular engineering.

### DFT calculations

**Fig. 4:**
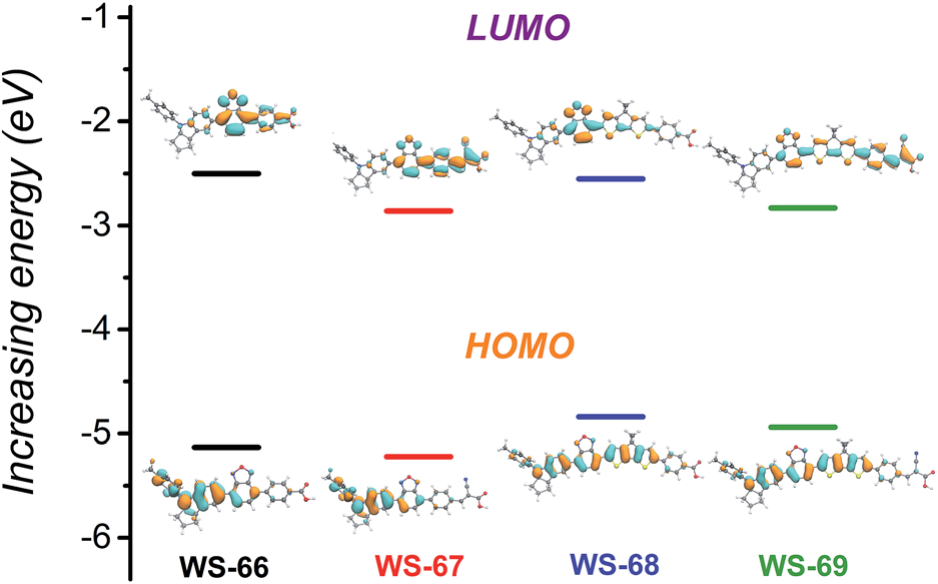
Calculated frontier molecular orbitals (HOMO and LUMO) and energy gaps for dyes **WS-66**, **WS-67**, **WS-68** and **WS-69**.

Density functional theory (DFT) calculations were employed using the hybrid B3LYP functional and the 6-31G(d) basis set (ESI†) to simulate the energy levels and electron distribution in the frontier orbitals.^21^ As presented in Fig. 4, the change in the calculated energy band gaps (Table S1†) is in agreement with the experimental data reducing from **WS-66** (2.63 eV) to **WS-69** (2.12 eV). For dyes **WS-66** and **WS-67**, the HOMO orbitals are mainly distributed on the indoline donor and BOD auxiliary acceptor, while the LUMO orbitals are largely delocalized on BOD, the benzene unit and the anchoring group. In comparison, the insertion of the CPDT moiety in **WS-68** and **WS-69** results in an extended delocalization of the HOMO and LUMO onto CPDT, thus enhancing the ICT character band, together with increasing the oscillator strength (Table S2†). All four sensitizers with either cyanoacetic acid or carboxylic acid acceptors display delocalized LUMO orbitals over the anchoring groups. Apparently, the well-overlapping HOMO and LUMO orbitals can facilitate electron migration from the donor to the anchoring group and injection into the TiO_2_ conduction band.^18^ Besides, the twisted dihedral angle between the BOD and benzene ring can be significantly relieved by the introduction of a co-planar CPDT unit (Table S3†). Especially for dye **WS-69**, the dihedral angles were minimized to 1.7° for BOD–CPDT and 10.6° for CPDT–benzene ring, which contributes to the convenient electron flow through the entire molecular skeleton.

### Enhanced *J*_SC_ from **WS-66** to **WS-69** accompanied by a decrease of *V*_OC_

**Fig. 5:**
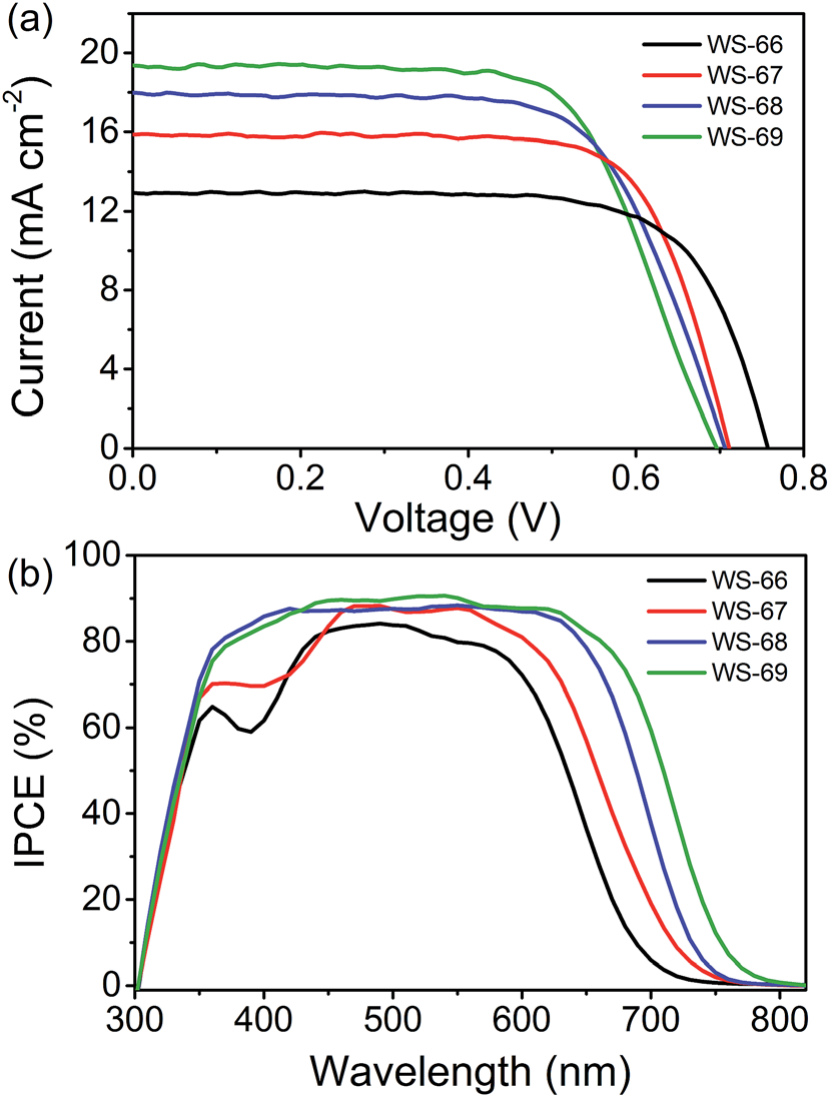
Current–voltage (*J*–*V*) curve (a) and IPCE spectra (b) of cell devices sensitized with dyes **WS-66**, **WS-67**, **WS-68** and **WS-69** under full AM 1.5 solar intensity.

Fig. 5 shows the photocurrent density–voltage (*J*–*V*) characteristic curves (a) of DSSCs fabricated with the prepared dyes using an iodine electrolyte containing 0.6 M of DPMII, 0.05 M of I_2_, 0.1 M of LiI, and 0.5 M of TBP in acetonitrile under AM 1.5 full sunlight (100 mW cm^–2^). The relevant parameters of the DSSCs are listed in Table 2. Detailed information regarding the DSSCs’ fabrication is included in the experimental section (ESI†). For the dye bath, 0.3 mM of dye was dissolved in chloroform/ethanol (4 : 1) without any co-adsorbent. Interestingly, the PCE values of these devices are increased in the order of **WS-66** (7.01%) < **WS-67** (8.25%) < **WS-68** (8.42%) < **WS-69** (9.03%), which can be attributed to the enhancement in *J*_SC_ from 12.97 to 15.91 to 17.73 to 19.39 mA cm^–2^. Compared to the devices sensitized by **WS-66** and **WS-68** with the carboxylic acid anchoring group, **WS-67** and **WS-69** bearing a stronger cyanoacetic acid group show the increased *J*_SC_ by 2.94 and 1.66 mA cm^–2^ due to a slightly improved photo-response. Meanwhile, an obvious decrease of 46 and 9 mV in the *V*_OC_ can also be seen, and mainly stems from the cyano group increasing the undesired backwards electron transfer. By incorporating CPDT into the skeleton of **WS-66** and **WS-67**, significant improvements in the *J*_SC_ of 4.76 and 3.48 mA cm^–2^ are obtained for the **WS-68** and **WS-69** sensitized cells owing to the greatly broadened light-harvesting and IPCE (Fig. 5b), albeit with a *V*_OC_ loss about 10 mV. Consequently, through rational molecular engineering, the optimal sensitizer, **WS-69**, realized the highest PCE of 9.03%, with the average parameters of *J*_SC_ = 19.39 mA cm^–2^, *V*_OC_ = 696 mV and FF = 0.67, outperforming the reference dye, **WS-66**(7.01%), as a result of broadening absorption and improving the near-infrared light-harvesting.

**Table 2:** Detailed photovoltaic parameters of cell devices sensitized with dyes **WS-66**, **WS-67**, **WS-68** and **WS-69** under full AM 1.5 solar intensity^*a*^

Dyes	*J*_SC_ (mA cm^–2^)	*V*_OC_ (mV)	FF	*η* (%)
**WS-66**	12.97 ± 0.02	757 ± 4	0.71 ± 0.02	7.01 ± 0.05
**WS-67**	15.91 ± 0.04	711 ± 3	0.73 ± 0.02	8.25 ± 0.07
**WS-68**	17.73 ± 0.03	705 ± 2	0.67 ± 0.01	8.42 ± 0.04
**WS-69**	19.39 ± 0.05	696 ± 2	0.67 ± 0.01	9.03 ± 0.05

^*a*^Average values are based on three replicate measurements.

The incident photon-to-current conversion efficiencies (IPCE) spectra, a function confirming the variation in *J*_SC_ for DSSCs, are shown in Fig. 5b. It can be found that the IPCE action spectra become broader from **WS-66** to **WS-69**, which is in line with the light-harvesting of the dye-loaded TiO_2_ films (Fig. S1†). In comparison with the carboxylic acid groups (**WS-66** and **WS-68**), dyes bearing cyanoacetic acid (**WS-67** and **WS-69**) show relatively broader IPCE spectra with higher plateaus. These behaviours are consistent with previously reported results from another group.^22^ Notably here, the optimal dye, **WS-69**, exhibits a high IPCE value exceeding 80% from 375 to 660 nm and an onset at 800 nm, along with no distinct valley over the whole visible wavelength. The enhancement in the IPCE spectra also demonstrates the significance of narrowing the band gap with suitable energy levels for DSSCs.

Meanwhile, we noticed that, despite the enhancement in *J*_SC_, the *V*_OC_ value for these dyes undesirably decreased from 757 mV for **WS-66** to 696 mV for **WS-69**. This probably results from the increased molecular size of **WS-69** combining the cyano unit in the anchoring group, resulting in a slightly tilted binding geometry and aggravated dye aggregation, then leading to faster charge recombination between the dye radical cation and electron in TiO_2_.^10–12^ It is deemed to be the “trade-off” effect between *J*_SC_ and *V*_OC_, which is hard to avoid in the molecular design of sensitizers.^9–13^ In this context, since the high *J*_SC_ with broad IPCE action spectra were obtained from molecular engineering, we try to further overcome the *V*_OC_ loss through a cosensitization strategy using a known high *V*_OC_ organic dye **WS-5** (Fig. S3 and Table S4†) for pursuing better photovoltaic performance. For comparison, two cosensitization couples of **WS-68**/**WS-5** and **WS-69**/**WS-5** were prepared and tested under the same conditions.

### Sequential cosensitization to prevent the “trade-off” effect between *J*_SC_ and *V*_OC_

**Fig. 6:**
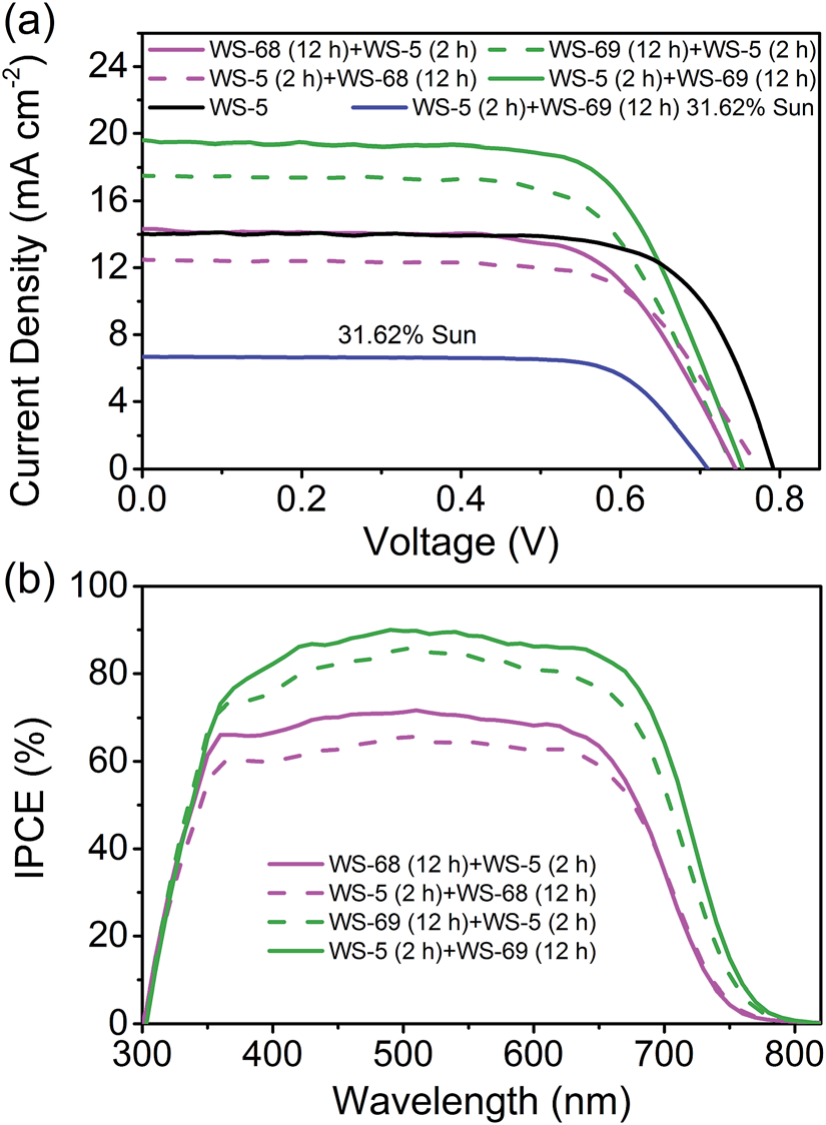
Current–voltage (*J*–*V*) curve (a) and IPCE spectra (b) of DSSCs made by cosensitization of **WS-68**/**69** with **WS-5**.

The cosensitized DSSCs of **WS-68**/**WS-69** with **WS-5** were made with the previous iodine electrolyte, and measured at an irradiation of simulated AM 1.5 sunlight. The *J*–*V* curves, IPCE spectra and corresponding photovoltaic parameters are presented in Fig. 6 and Table 3. We focused on probing the effect of the cosensitization sequence on their photovoltaic performances. The cosensitized TiO_2_ electrode was first dipped in a solution of **WS-68**/**WS-69** for 12 h, then dipped in a solution of **WS-5** for 2 h, indicated as **WS-68** (12 h) + **WS-5** (2 h) and **WS-69** (12 h) + **WS-5** (2 h), respectively. Unexpectedly, both of the cosensitization couples show a decreased PCE due to the decreased *J*_SC_ (14.08 and 17.36 mA cm^–2^) compared to their single dye (17.73 and 19.39 mA cm^–2^), which can be reflected in their relatively low IPCE values (Fig. 6b). However, it is promising that cosensitization leads to an obvious increase in *V*_OC_ up to 740 mV. Next, the reversed co-adsorption sequence was explored, in which the TiO_2_ electrode was first dipped in a **WS-5** solution for 2 h, then dipped in a **WS-68**/**WS-69** solution for 12 h, indicated as **WS-5** (2 h) + **WS-68** (12 h) and **WS-5** (2 h) + **WS-69** (12 h), respectively. Interestingly, totally disparate cell behaviours were obtained for these two cosensitization couples. For the **WS-5** (2 h) + **WS-68** (12 h) cosensitization, the cell performances are extremely poor with a PCE of 6.56% due to the sharp reduction in photocurrent to 12.51 mA cm^–2^. However, the *V*_OC_ here for cosensitization is boosted to 774 mV, which is even close to the *V*_OC_ of single **WS-5** (792 mV). We speculate that a large adsorption replacement of **WS-68** by **WS-5** exists which dominated the cell’s behaviour (discussed in the adsorption amount experiment section). Apparently, the cosensitization sequence can exert a tremendous influence on the cell’s performance, especially for the *V*_OC_ value. In contrast, in the case of **WS-5** (2 h) + **WS-69** (12 h), surprisingly, the cosensitized cells exhibit a significantly enhanced PCE reaching 10.09%, along with the photovoltaic parameters *J*_SC_ = 19.56 mA cm^–2^, *V*_OC_ = 753 mV and FF = 0.68. It is remarkable that synergistic improvements both in the photocurrent and photovoltage can be obtained *via* cosensitization with respect to the single **WS-69**. Note that the *V*_OC_ value increases by 57 mV from 696 to 753 mV for the cosensitized cells. Also, to meet the requirement of indoor DSSCs’ applications, the best device (**WS-5** (2 h) + **WS-69** (12 h)) was tested at a weaker light irradiation. Strikingly, the optimal cells achieve a notable PCE of 11.12% under 31.62% sunlight illumination owing to the high FF. To the best of our knowledge, this is the first time that the efficiency of a pure metal-free organic dye with an iodine electrolyte exceeds 11% even under relatively weak light irradiation. Indeed, the leading cosensitized cells (**WS-5** (2 h) + **WS-69** (12 h)) display an impressive IPCE action spectrum which is even stronger than the single **WS-69** based device, along with a high value beyond 80% from 385 to 675 nm and an onset at 800 nm. The enhanced IPCE behaviour is probably attributed to the improved electron injection efficiency due to the adsorption of **WS-5**. The calculated photocurrent from the IPCE spectrum is in good agreement with the measured *J*_SC_. Indeed, we have successfully overcome the “trade-off” effect by carefully exploring sequential cosensitization. In contrast with the previous cosensitization strategy mostly focused on the compensation of light-harvesting, we here propose a novel cosensitization architecture, in which the large molecular-sized high *J*_SC_ dye, **WS-69**, takes charge of broadening the light-harvesting region to generate a high photocurrent while the small molecular-sized high *V*_OC_ dye, **WS-5**, is responsible for retarding charge recombination to generate a high photovoltage (discussed in the electrochemical impedance spectroscopy (EIS) analysis section). The cosensitization of **WS-69** with **WS-5** with different molecular sizes can form a compact and synergetic sensitized layer on the TiO_2_ surface to effectively block backwards electron transfer between the injected electrons and the redox species. Moreover, the choice of co-adsorption dye and the co-adsorption sequence are demonstrated to be very critical for the cell behaviours, especially for the cosensitization of dye with different anchoring group which deserves to be more carefully studied to achieve higher efficiency.

**Table 3:** Detailed photovoltaic parameters of cell devices fabricated with cosensitization of **WS-68**/**WS-69** with **WS-5** under different light intensities^*d*^

Dyes	*J*_SC_ (Cal)^*c*^ (mA cm^–2^)	*J*_SC_ (mA cm^–2^)	*V*_OC_ (mV)	FF	*η* (%)
**WS-68** (12 h) + **WS-5** (2 h)^*a*^	13.98	14.08 ± 0.06	746 ± 3	0.67 ± 0.01	7.67 ± 0.06
**WS-69** (12 h) + **WS-5** (2 h)^*a*^	17.19	17.36 ± 0.03	744 ± 2	0.67 ± 0.02	8.66 ± 0.02
**WS-5** (2 h) + **WS-68** (12 h)^*a*^	12.93	12.51 ± 0.05	774 ± 2	0.68 ± 0.02	6.56 ± 0.05
**WS-5** (2 h) + **WS-69** (12 h)^*a*^	18.66	19.56 ± 0.03	753 ± 3	0.68 ± 0.01	10.09 ± 0.02
**WS-5** (2 h) + **WS-69** (12 h)^*b*^	—	6.67 ± 0.04	710 ± 2	0.74 ± 0.01	11.12 ± 0.03
**WS-5** ^*a*^	—	14.05 ± 0.03	792 ± 2	0.72 ± 0.01	8.01 ± 0.03

^*a*^100% solar intensity irradiation.

^*b*^31.62% solar intensity irradiation.

^*c*^Calculated *J*_SC_ value from the IPCE spectrum.

^*d*^Average values are based on three replicate measurements.

### Cosensitization brings forth long-lived electron lifetime

**Fig. 7:**
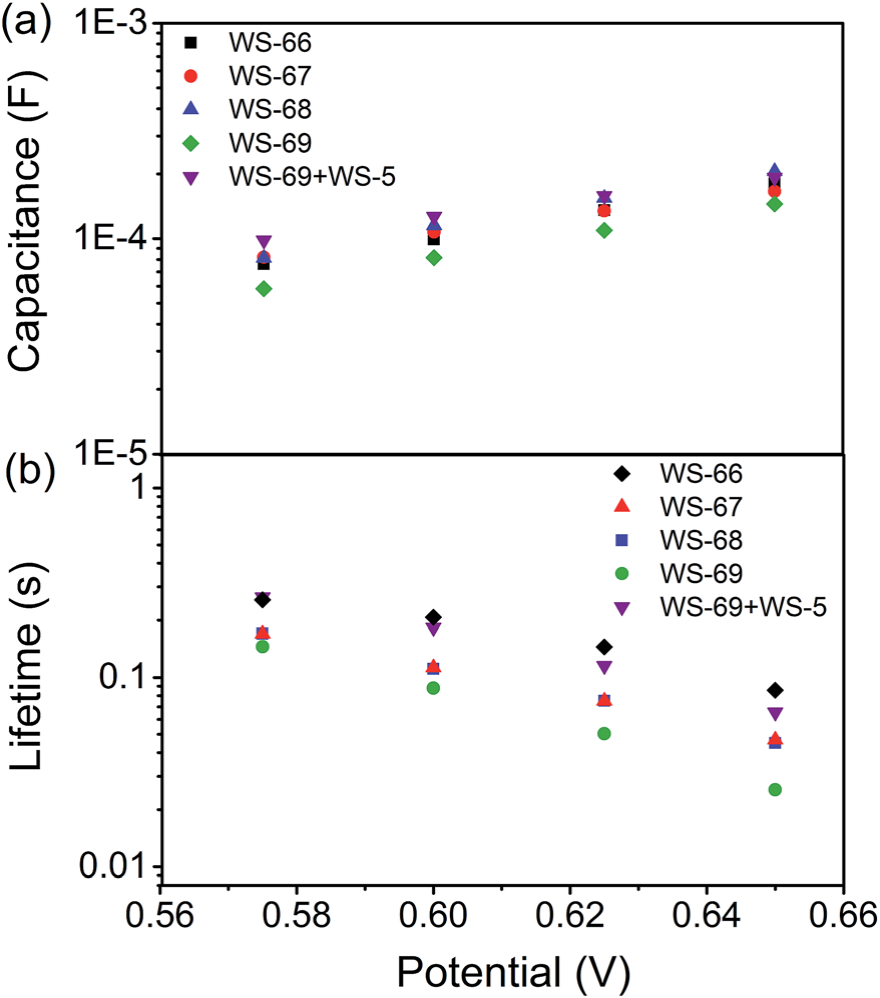
TiO_2_ capacitance (a) and electron lifetime (b) as a function of potential based on **WS-66**, **WS-67**, **WS-68**, **WS-69** and cosensitization of **WS-5** with **WS-69**.

To get insight into the different *V*_OC_ values resulting from modulation of the energy level and cosensitization, electrochemical impedance spectroscopy (EIS) analysis was applied to the DSSCs based on single **WS-66**, **WS-67**, **WS-68**, **WS-69** and the optimal cosensitized couple (**WS-5** (2 h) + **WS-69** (12 h)). Generally, the *V*_OC_ is determined by the quasi-Fermi level of TiO_2_ and the redox couple in the electrolyte.^23^ Since the electrolyte components used in this work are identical, the variation in *V*_OC_ can be attributed to the change of the quasi-Fermi level of TiO_2_, which is mainly influenced by the conduction band position and free charge density in TiO_2_. As shown in Fig. 7a, nearly no obvious change in the capacitance can be found for the four individual dyes, while co-adsorption of **WS-69** with **WS-5** brings about a slightly upwards shifted conduction band edge reflected in its higher capacitance. Then, the electron lifetime as a function of free charge density was further measured and plotted in Fig. 7b. At a given bias voltage, the lifetime for DSSCs sensitized by individual dyes decreases in the order **WS-66** > **WS-67** > **WS-68** > **WS-69**, which is in line with the *V*_OC_ change, suggestive of the aggravated charge recombination process between electrons injected into TiO_2_ with an iodine electrolyte or oxidized dyes. Notably, upon co-adsorption of **WS-69** with **WS-5**, the cosensitized device exhibits a significantly prolonged electron lifetime even close to that of **WS-66**, indicative of its slowed charge recombination rate. Here we consider that the large-sized molecule (**WS-69**) cosensitized with a small-sized molecule (**WS-5**) can form a compact and synergetic sensitized layer on the TiO_2_ surface, not only suppressing dye aggregation and weakening intermolecular interactions but also largely removing the charge recombination channels between the injected electrons and the iodine electrolyte. Accordingly, it can be concluded that the dramatic increase of 57 mV in the *V*_OC_ value for the cosensitized cells is a cumulative effect caused by the upward shifted conduction band and enhanced deterring of the charge recombination process with prolonged electron lifetime. EIS analysis further demonstrates that the cosensitization strategy is a feasible method for improving the *V*_OC_ for the development of DSSCs.

### The cyano group benefits the binding of dyes onto TiO_2_ nanocrystals

**Fig. 8:**
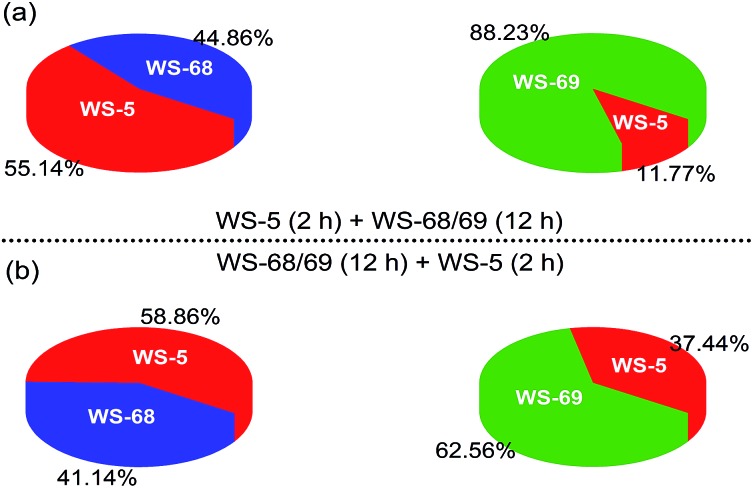
Pie charts showing the adsorption proportions of dyes **WS-5**, **WS-68** and **WS-69** in the cosensitization strategy on 16 μm TiO_2_ films.

Since the only difference between **WS-68** and **WS-69** is the anchoring group, we try to investigate their adsorption ability as well as dye composition change in the cosensitization process. The adsorption amounts (Table S5†) of the individual dye and cosensitization were measured from UV-Vis spectra of a desorption dye solution (0.1 M NaOH in THF/H_2_O (1/1)) and are presented in Fig. 8. Compared to dye **WS-68** which bears a carboxylic acid anchoring group (1.60 × 10^–7^ mol cm^–2^), **WS-69** bearing a cyanoacetic acid group shows a larger dye-loading amount of 2.18 × 10^–7^ mol cm^–2^ in a 12 h dipping time. Meanwhile, **WS-5** can also achieve a considerable dye-loading amount of 1.20 × 10^–7^ mol cm^–2^ in just 2 h probably due to its small molecule size. Upon co-adsorption, when the electrode was first dipped into the **WS-5** solution for 2 h, then dipped into **WS-68**/**WS-69** for 12 h, all of the three dyes show decreased adsorption amounts compared to their individual adsorption, indicative of the serious competitive adsorption among the co-adsorbed dyes. Meanwhile, although **WS-68** has undergone a longer adsorption time of 12 h than of **WS-5** (2 h), the proportion of **WS-68** in all dye-loading (**WS-5** + **WS-68**) is just 44.86%, even less than that of **WS-5** (55.14%). Thereby, we can understand from this that the high *V*_OC_ close to that of **WS-5** in this cosensitized couple mainly results from the advantageous adsorption amount of **WS-5**. However, in case of the co-adsorption of **WS-5** and **WS-69**, the dye-loading amount of **WS-69** (88.23%) is much higher than **WS-5** (11.77%) and nearly double that of **WS-68** (44.86%) under the same adsorption conditions. Hence, **WS-5** working as a benchmark can identify the discrepant binding strength of the two anchoring units with TiO_2_. We can conclude that the cyanoacetic acid anchoring group possesses a stronger adsorption ability than the carboxylic acid group. Then the reversed adsorption sequence was also carried out, and similar results can be obtained in that the ratio of **WS-68** is still less than **WS-5** and **WS-69**, further supporting the relative weaker binding strength of carboxylic acid with TiO_2_. Besides, the **WS-5** and **WS-69** co-adsorption couple exhibits a greater total adsorption amount (2.25 × 10^–7^ and 2.64 × 10^–7^ mol cm^–2^) than the **WS-5** and **WS-68** co-adsorption couple (1.48 × 10^–7^ and 1.70 × 10^–7^ mol cm^–2^), which is thought to be a key factor of their different cells’ behaviours.

### High-stability afforded by rational molecular engineering

Stability is considered to be a crucial requirement for further solar cell applications. Thereby, we first tested the photo-stability of four sensitizers with different molecular structures. According to the method reported by Katoh and coworkers,^24^ the most unstable state of sensitizers is their oxidized state. Sensitizers are required to be stable in their cation state for at least 10 s to be able to realize a 10 year operation cycle. To shorten the experiment period, the absorbance of a dye-loaded TiO_2_ film without a redox electrolyte was measured under simulated AM 1.5G (15 and 30 min), in which the dye regeneration process occurs in the millisecond time range, taking 10^4^ to 10^3^ times longer than that in a complete DSSC device (100 ns to 1 μs).

**Fig. 9:**
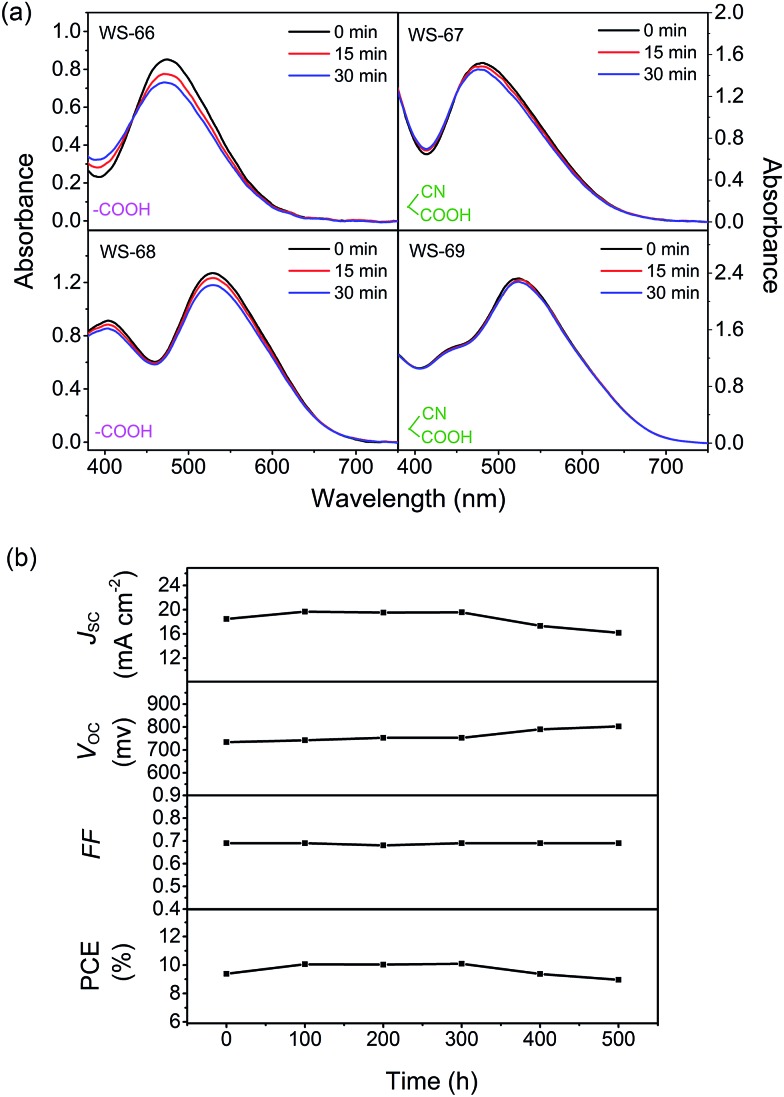
Dyes **WS-66**, **WS-67**, **WS-68** and **WS-69** adsorbed on TiO_2_ films before and after light irradiation for 15 and 30 min (a), evolution of photovoltaic performance parameters for DSSCs based on cosensitization of **WS-69** with **WS-5** under visible-light soaking (b).

As depicted in Fig. 9a, **WS-66** shows obvious attenuation in absorbance after light irradiation, suggesting its relatively weak stability. Incorporation of a CPDT unit can remarkably improve the dye stability with reduced attenuation after illumination as reflected in **WS-68**, which can be ascribed to the more effective separation of the HOMO orbital on the CPDT unit as illustrated in the DFT calculation. On the other hand, it can be found that **WS-67** and **WS-69** with cyanoacetic acid acceptors display enhanced stability compared with **WS-66** and **WS-68** with carboxylic acid acceptors,^25^ which can be attributed to two factors: (i) a more stabilized LUMO orbital resulting from the electron-withdrawing cyano group, and (ii) stronger binding strength of cyanoacetic acid with TiO_2_ nanocrystals as demonstrated in the adsorption amount experiment. Apparently, **WS-69** exhibits the strongest photo-stability with no obvious shift and decay after 30 min irradiation, which is considered to be better than that of **MK-2**.^24^ Afterwards, the long-term durability of cell devices based on the leading dye **WS-69** and cosensitization (**WS-5** (2 h) + **WS-69** (12 h)) was also tested with a liquid iodine electrolyte (Fig. S4† and 9b). As a result, the PCE of the cosensitized cell remained at 96% of its initial value after 500 h aging. These results unambiguously corroborate the superior stability of dye **WS-69**, which is afforded by rational molecular engineering.

## Conclusions

We have focused on the detrimental “trade-off” effect between photocurrent and photovoltage, and developed four novel benzoxidiazole based D–A–π–A metal-free organic dyes with different combinations of π-spacer units and anchoring-acceptor groups (**WS-66**, **WS-67**, **WS-68** and **WS-69**). By stepwise tuning of the LUMO and HOMO levels, the optimal sensitizer **WS-69** exhibits the narrowest energy band gap and broadest absorption spectrum, along with the highest photocurrent of up to 19.39 mA cm^–2^ but suffers from an inferior photovoltage of 696 mV. Sequential cosensitization with a small molecular-sized high *V*_OC_ dye **WS-5** was demonstrated to effectively remedy the *V*_OC_ loss for dye **WS-69** and distinctly prevent the “trade-off” effect between *J*_SC_ and *V*_OC_. The leading cosensitized cells exhibit an excellent efficiency of 10.09% under one sun irradiation, and 11.12% under 0.3 sun irradiation. To the best of our knowledge, this is the first time that the efficiency of a pure metal-free organic dye with an iodine electrolyte exceeds 11% even under relatively weak light irradiation. We further propose a novel cosensitization architecture, in which the large molecular-sized, high *J*_SC_ dye **WS-69** takes charge of broadening the light-harvesting region to generate a high photocurrent while the small molecular-sized, high *V*_OC_ dye **WS-5** is responsible for retarding charge recombination to generate a high photovoltage. Moreover, the adsorption amount and photo-stability studies suggest that the cyano group in the anchoring acceptor is important for the stability since it is beneficial towards decreasing the LUMO levels and enhancing the binding of dyes onto the TiO_2_ nanocrystals.
