# Supplementary material for: Molecular engineering and sequential cosensitization for preventing the “trade-off” effect with photovoltaic enhancement
Source: Chem Sci. 2016 Nov 17;8(3):2115–24. doi: 10.1039/c6sc03938c (PMC5407262; doi:10.1039/c6sc03938c)
Supplement: Supplementary file 1 [file SC-008-C6SC03938C-s001.pdf]

Electronic Supplementary Material (ESI) for Chemical Science.
This journal is © The Royal Society of Chemistry 2016

Electronic Supplementary Information (ESI)

Molecular engineering and sequential cosensitization for preventing "trade-off" effect with photovoltaic enhancement†

Weiwei Zhang,[a] Yongzhen Wu,[a] Xin Li,[b] Erpeng Li,[a] Xiongrong Song,[a] Huiyun Jiang,[a] Chao Shen,[a] Hao Zhang,[a] He Tian[a] and Wei-Hong Zhu*[a]

[a]*Shanghai Key Laboratory of Functional Materials Chemistry, Key Laboratory for Advanced Materials and Institute of Fine Chemicals, Collaborative Innovation Center for Coal Based Energy (i-CCE), School of Chemistry and Molecular Engineering, East China University of Science and Technology, Shanghai 200237, P. R. China; E-mail: whzhu@ecust.edu.cn*

[b] *Division of Theoretical Chemistry and Biology, School of Biotechnology, KTH Royal Institute of Technology, SE-10691 Stockholm, Sweden.*

Contents

1. **Materials and reagents……………………….……………………………………….S2**
2. **Synthesis..S2**
3. **DSSCs fabrication..S5**
4. **Measurement equipment and apparatus……………………………………………..S6**
5. **Light harvesting efficiency of dyes WS-66, WS-67, WS-68 and WS-69…………………S7**
6. **Computational details……………………………………………………………….S8**
7. **Molecular structures of dye WS-5………...…………………………………………..S9**
8. **Optical and electrochemical data for dyes WS-5……………………………………S9**
9. **Adsorption amount of individual dye and cosensitization………………………………S10**
10. **Evolutions of DSSCs based on single WS-69 under visible-light soaking…………..….S10**
11. **^{1}H, ^{13}C NMR and HR-MS of intermediates and tarteted sensitizers..............................S11**

1. Materials and reagents.

All chemicals and solvents used in this work were of reagent grade and were obtained from J&K Scientific, Adamas Reagent Co Ltd and so on. Tetrahydrofuran was dried by sodium under argon atmosphere before use. (4-formylphenyl)boronic acid **(4)** and 4,4-dioctyl-4*H*-cyclopenta[2,1-*b*:3,4-*b*']dithiophene **(6)** were purchased from Derthon Co Ltd. 4-bromo-7-(4-(*p*-tolyl)-1,2,3,3*a*,4,8*b*-hexahydrocyclopenta[*b*]indol-7-yl)benzo[*c*][1,2,5]oxadiazole **(1)**,[S1] butyl 4-(4,4,5,5-tetramethyl-1,3,2-dioxaborolan-2-yl)benzoate **(2)**[S2] were synthesized according to the previous literature.

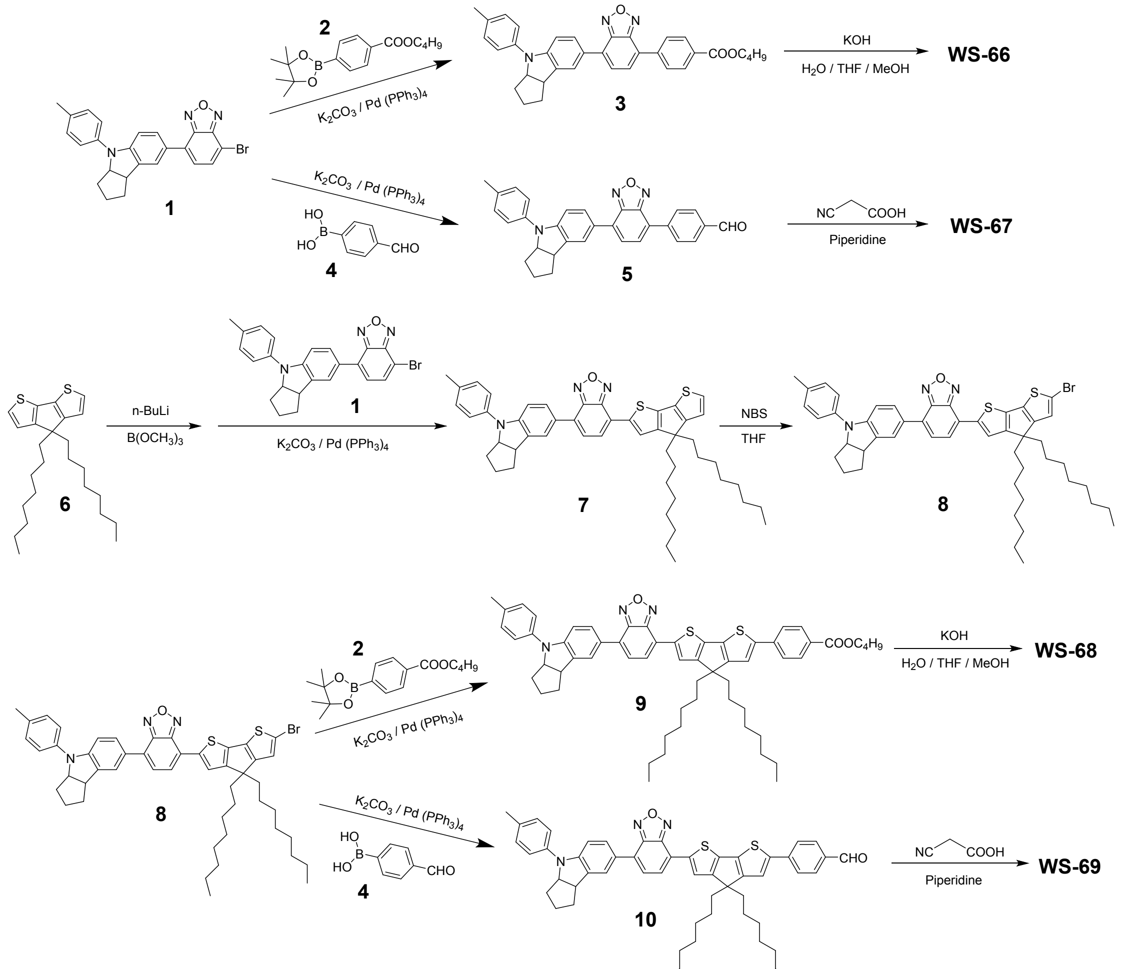

Scheme 1 Synthetic route for sensitizers **WS-66**, **WS-67**, **WS-68** and **WS-69**.

2. Synthesis

Synthesis of compound **3**. The compound **1** (300 mg, 0.67 mmol), **2** (260 mg, 0.85 mmol) were mixed with 2 M K_2CO_3 (15 mL) and Pd $(PPh_3)_4$ (100 mg) in THF (40 mL), the reaction mixture was heated to 80 °C and stirred for 8 h. After completion of the reaction, the organics were evaporated and the residue was dissolved in CH_2Cl_2 and washed with brine, dried over anhydrous Na_2SO_4, evaporated to remove the solvent. The crude product was then purified by column chromatography on silica gel (petroleum ether : CH_2Cl_2 = 3 : 1) to afford a red oil **3** (203 mg, 55.7%). ^{1}H NMR (400 MHz, $CDCl_3$, δ, ppm): 8.16 (d, J = 8.6 Hz, 2 H), 8.08 (d, J = 8.6 Hz, 2 H), 7.84 (s, 1 H), 7.78 (dd, J_1 = 8.4 Hz, J_2 = 1.9 Hz, 1 H), 7.65 (d, J = 7.4 Hz, 1 H), 7.53 (d, J = 7.4 Hz, 1 H), 7.21 (d, J = 8.7

Hz, 2 H), 7.17 (d, J = 8.6 Hz, 2 H), 6.95 (d, J = 8.4 Hz, 1 H), 4.85 (m, 1 H), 4.36 (t, J = 6.6 Hz, 2 H), 3.88 (m, 1 H), 2.34 (s, 3 H), 2.13-2.06 (m, 1 H), 1.96-1.90 (m, 2 H), 1.81-1.74 (m, 3 H), 1.71-1.64 (m, 1 H), 1.61-1.55 (m, 1 H), 1.54-1.48 (m, 2 H), 1.00 (t, J = 7.4 Hz, 3 H). ^{13}C NMR (100 MHz, $CDCl_3$, δ, ppm): 166.34, 149.41, 149.35, 149.16, 139.76, 135.76, 132.22, 130.26, 130.05, 129.97, 129.90, 128.38, 127.95, 127.21, 125.28, 124.92, 124.71, 120.67, 107.41, 69.38, 65.01, 45.30, 35.33, 33.60, 30.82, 24.42, 20.88, 19.33, 13.83. HRMS-ESI (*m/z*): $[M + H]^+$ Calcd. for ($C_{35}H_{34}N_3O_3$), 544.2600, found: 544.2601.

Synthesis of **WS-66**. Under argon, compound **3** (203 mg, 0.37 mmol) and KOH (500 mg, 8.91 mmol) were dissolved in MeOH (10 mL), H_2O (10 mL) and THF (40 mL). The solution was heated to 70°C and stirred for 2 h. The organics were evaporated and the residue was dissolved in CH_2Cl_2 and washed with brine, dried over anhydrous Na_2SO_4, evaporated to remove the solvent. The crude product was then purified by column chromatography on silica gel (CH_2Cl_2 : MeOH = 10 : 1) to afford a red solid **WS-66** (137 mg, 75.9%).^{1}H NMR (400 MHz, THF-d_8, δ, ppm): 8.10 (d, J = 8.4 Hz, 2 H), 8.04 (d, J = 8.5 Hz, 2 H), 7.85 (s, 1 H), 7.78 (d, J = 7.7 Hz, 2 H), 7.62 (d, J = 7.4 Hz, 1 H), 7.13 (d, J = 8.4 Hz, 2 H), 7.06 (d, J = 8.3 Hz, 2 H), 6.84 (d, J = 8.5 Hz, 1 H), 4.82 (m, 1 H), 3.81 (m, 1 H), 1.96 (s, 3 H), 1.80-1.82 (m, 1 H), 1.69-1.72 (m, 2 H), 1.57-1.66 (m, 1 H), 1.53-1.55 (m, 1 H), 1.41-1.47 (m, 1 H). ^{13}C NMR (100 MHz, THF-d_8, δ, ppm): 164.58, 147.46, 147.44, 147.30, 137.95, 133.73, 129.96, 128.27, 128.05, 127.74, 127.65, 127.61, 126.44, 125.84, 123.49, 123.01, 122.85, 122.77, 122.64, 118.66, 105.16, 67.37, 52.04, 33.33, 31.52, 27.80, 18.05. HRMS-ESI (*m/z*): $[M + H]^+$ Calcd. for ($C_{31}H_{26}N_3O_3$), 488.1974, found: 488.1978.

Synthesis of compound **5**. The compound **5** was synthesized in a similar manner to that for compound **3** (192 mg, 64.6%). ^{1}H NMR (400 MHz, $CDCl_3$, δ, ppm): 10.07 (s, 1 H), 8.20 (d, J = 8.0 Hz, 2 H), 8.00 (d, J = 8.1 Hz, 2 H), 7.86 (s, 1 H), 7.80 (d, J = 8.4 Hz, 1 H), 7.71 (d, J = 7.4 Hz, 1 H), 7.56 (d, J = 7.4 Hz, 1 H), 7.22 (d, J = 8.3 Hz, 2 H), 7.18 (d, J = 8.3 Hz, 2 H), 6.96 (d, J = 8.4 Hz, 1 H), 4.88 (t, J = 7.0 Hz, 1 H), 3.90 (t, J = 8.5 Hz, 1 H), 2.35 (s, 3 H), 2.15-2.05 (m, 1 H), 1.94 (m, 2 H), 1.84-1.75 (m, 1 H), 1.68 (s, 1 H), 1.59-1.54 (m, 1 H). ^{13}C NMR (100 MHz, $CDCl_3$, δ, ppm): 191.75, 149.59, 149.35, 149.09, 141.36, 139.56, 135.89, 135.81, 132.36, 130.68, 130.41, 130.20, 129.92, 128.56, 128.49, 125.15, 124.75, 124.56, 124.47, 120.77, 107.40, 69.42, 45.28, 35.35, 33.60, 24.41, 20.88. HRMS-ESI (*m/z*): $[M + H]^+$ Calcd. for ($C_{31}H_{26}N_3O_2$), 472.2025, found: 472.2029.

Synthesis of **WS-67**. Under argon, compound **5** (192 mg, 0.41 mmol) and cyanoacetic acid (100 mg, 1.18 mmol) were dissolved in acetonitrile (30 mL) and refluxed for 8 h. Then the solvent was removed under reduced pressure. The residue was dissolved in CH_2Cl_2 and washed with brine, dried over anhydrous Na_2SO_4, evaporated to remove the solvent and purified by column chromatography on silica gel (CH_2Cl_2 : MeOH = 10 : 1) to afford a red solid **WS-67** (152 mg, 68.8%). ^{1}H NMR (400 MHz, DMSO-d_6, δ, ppm): 8.22 (d, J = 6.9 Hz, 2 H), 8.07 (m, 3 H), 8.01 (d, J = 7.0 Hz, 1 H), 7.92 (s, 1 H), 7.86 (m, 2 H), 7.25 (m, 2 H), 7.22 (m, 2 H), 6.95 (d, J = 8.4 Hz, 1 H), 4.95 (m, 1 H), 3.91 (m, 1 H), 2.30 (s, 3 H), 2.09 (s, 1 H), 1.77-1.84 (s ,3 H), 1.42 (s, 1 H), 1.28 (s, 1 H). ^{13}C NMR (100 MHz, DMSO-d_6, δ, ppm):163.81, 148.78, 148.65, 148.48, 147.46, 139.03, 136.96, 135.51, 133.04, 131.34, 130.70, 129.91, 129.79, 128.48, 128.27, 128.15, 126.17, 124.29, 124.06, 123.55, 120.05, 118.80, 112.68, 106.78, 68.41, 43.40, 34.89, 32.94, 22.04, 21.71. HRMS-ESI (*m/z*): $[M + H]^+$ Calcd.

for ($C_{34}H_{27}N_4O_3$), 539.2083, found: 539.2081.

Synthesis of compound **7**. Under argon, to a solution of compound **6** (900 mg, 2.23 mmol) in dry THF (20 mL) was added n-BuLi (1.12 mL, 2.69 mmol) dropwise at -78 °C. The resulting solution was stirred for 1 h before adding $B(OCH_3)_3$ (0.38 mL, 3.35 mmol). The stirring was maintained at -78 °C for another 3 h for next Suzuki reaction without any purification. Under argon, the previous prepared mixture was reacted with compound **1** (995 mg, 2.23 mmol) with 2 M K_2CO_3 (15 mL) and $Pd(PPh_3)_4$ (100 mg) in THF (40 mL) for 6 h. After cooling to the room temperature, the organics solvent were evaporated and the residue was dissolved in CH_2Cl_2 and washed with brine for two times, dried over anhydrous Na_2SO_4, evaporated to remove the solvent. The crude product was then purified by column chromatography on silica gel (petroleum ether : CH_2Cl_2 = 4 : 1) to afford a purple solid **7** (926 mg, 54.2%).^{1}H NMR (400 MHz, $CDCl_3$, δ, ppm): 8.01 (s, 1 H), 7.86 (s, 1 H), 7.79 (d, J = 8.4 Hz, 1 H), 7.59 (d, J = 7.5 Hz, 1 H), 7.49 (d, J = 7.5 Hz, 1 H), 7.24 (m, 1 H), 7.23 (d, J = 5.9 Hz, 2 H), 7.18 (d, J = 8.4 Hz, 2 H), 6.98 (m, 2 H), 4.88 (m, 1 H), 3.93 (m, 1 H), 2.35 (s, 3 H), 2.09 (m, 1 H), 1.98 (m, 2 H), 1.91 (m, 4 H), 1.80 (m, 1 H), 1.69 (m, 1 H) 1.60 (m, 1 H), 1.19 (m, 20 H), 0.99 (m, 4 H), 0.82 (m, 6 H). ^{13}C NMR (100 MHz, $CDCl_3$, δ, ppm):159.50, 159.21, 149.21, 148.99, 148.22, 139.83, 138.27, 137.79, 136.33, 135.72, 132.04, 129.88, 127.97, 127.45, 126.08, 125.80, 125.23, 125.01, 124.48, 122.99, 121.80, 121.37, 120.54, 107.51, 69.37, 53.97, 45.36, 37.78, 35.29, 33.65, 31.83, 30.03, 29.36, 29.28, 24.58, 24.44, 22.64, 20.87, 14.10. HRMS-ESI (*m/z*): $[M + H]^+$ Calcd. for ($C_{49}H_{57}N_3OS_2$), 768.4201, found: 768.4205.

Synthesis of compound **8**. Compound **7** (526 mg, 0.68 mmol) was dissolved in dry THF (30 mL) at the temperature of 0 °C. NBS (145 mg, 0.81 mmol) was also dissolved in dry THF (10 mL) and added dropwise. The mixture was allowed to warm to room temperature and keep stirring for 5 h. The solvent was evaporated and the residue was kept for next Suzuki reaction without purification.

Synthesis of compound **9**. The compound **9** was synthesized in a similar manner to that for compound **3** (157 mg, 53.6%). ^{1}H NMR (400 MHz, $CDCl_3$, δ, ppm): 8.05 (s, 1 H), 8.03 (d, J = 3.2 Hz, 2 H), 7.87 (s, 1 H), 7.79 (dd, J_1 = 8.4 Hz, J_2 = 1.7 Hz, 1 H), 7.68 (d, J = 8.4 Hz, 2 H), 7.61 (d, J = 7.5 Hz, 1 H), 7.50 (d, J = 7.5 Hz, 1 H), 7.33 (s, 1 H), 7.23 (d, J = 8.6 Hz, 2 H), 7.19 (d, J = 8.5 Hz, 2 H), 6.98 (d, J = 8.4 Hz, 1 H), 4.88 (m, 1 H), 4.34 (t, J = 6.6 Hz, 2 H) 3.93 (m, 1 H), 2.36 (s, 3 H), 2.11 (m, 1 H), 1.94-1.97 (m, 6 H), 1.78 (m, 2 H), 1.69 (m, 1 H) 1.50 (m, 2 H), 1.25 (m, 5 H), 1.16-1.21 (m, 20 H), 1.00 (m, 4 H), 0.80 (m, 6 H). ^{13}C NMR (100 MHz, $CDCl_3$, δ, ppm): 166.37, 160.07, 159.83, 149.19, 149.10, 148.18, 144.07, 139.79, 139.44, 139.26, 137.50, 135.74, 132.13, 130.31, 129.88, 128.67, 128.03, 127.76, 125.70, 125.28, 125.15, 124.67, 124.50, 122.78, 121.12, 120.60, 119.07, 107.50, 69.40, 64.89, 54.47, 45.35, 37.83, 35.29, 33.64, 31.80, 30.82, 30.01, 29.71, 29.34, 29.27, 24.61, 24.43, 22.61, 20.85, 19.32, 14.06, 13.82. HRMS-ESI (*m/z*): $[M + H]^+$ Calcd. for ($C_{60}H_{70}N_3O_3S_2$), 944.4859, found: 944.4858.

Synthesis of compound **WS-68**. The **WS-68** was synthesized in a similar manner to that for **WS-66** (91 mg, 89.6 %). ^{1}H NMR (400 MHz, THF-d_8, δ, ppm): 10.74 (s, 1 H), 8.00 (s, 1 H), 7.91 (d, J = 8.2 Hz, 2 H), 7.86 (s, 1 H), 7.78 (d, J = 8.4 Hz, 1 H), 7.66 (d, J = 8.1 Hz, 2 H), 7.63 (s, 1 H), 7.56 (d, J = 7.5 Hz, 1 H), 7.50 (s, 1 H), 7.14 (d, J = 8.3 Hz, 2 H), 7.07 (d, J = 7.8 Hz, 2 H), 6.85 (d, J = 8.4 Hz, 1 H), 4.82 (m, 1 H), 3.82 (m, 1 H), 2.21 (s, 3 H), 2.02 (m, 1 H), 1.93 (m, 4 H), 1.83 (m, 2

H), 1.71 (m, 1 H), 1.55 (m, 1 H), 1.44 (m, 1 H), 1.07-1.18 (m, 20 H), 0.97 (m, 4 H), 0.69 (m, 6 H). ^{13}C NMR (100 MHz, THF-d$_8$, *δ*, ppm): 166.18, 160.10, 159.51, 149.03, 149.00, 148.11, 144.45, 139.89, 139.52, 138.95, 137.72, 137.16, 135.58, 131.71, 130.26, 129.59, 129.25, 127.97, 127.65, 125.53, 125.44, 125.15, 124.34, 124.29, 122.40, 120.74, 120.41, 119.32, 107.10, 69.20, 54.36, 45.31, 37.78, 35.17, 33.41, 31.79, 30.01, 29.67, 29.26, 22.49, 19.90, 13.39. HRMS-ESI (*m/z*): [M + H]$^+$ Calcd. for ($C_{56}H_{62}N_3O_3S_2$), 888.4233, found: 888.4230.

Synthesis of compound **10**. The compound **10** was synthesized in a similar manner to that for compound **3** (162 m, 68.8%). ^{1}H NMR (400 MHz, CDCl$_3$, *δ*, ppm): 9.99 (s, 1 H), 8.02 (s, 1 H), 7.89 (m, 3 H), 7.78 (m, 3 H), 7.62 (d, *J* = 7.0 Hz, 1 H), 7.51 (d, *J* = 7.4 Hz, 1 H), 7.38 (s, 1 H), 7.23 (d, *J* = 8.0 Hz, 2 H), 7.19 (d, *J* = 8.4 Hz, 2 H), 6.99 (d, *J* = 8.6 Hz, 1 H), 4.89 (m, 1 H), 3.93 (m, 1 H), 2.36 (s, 3 H), 2.11 (m, 1 H), 1.96 (m, 6 H), 1.81 (m, 1 H), 1.69 (m, 1 H) 1.59 (m, 1 H), 1.13-1.16 (m, 20 H), 1.04 (m, 4 H), 0.80-0.82 (m, 6 H). ^{13}C NMR (100 MHz, CDCl$_3$, *δ*, ppm): 191.31, 160.14, 149.19, 149.15, 148.17, 143.52, 140.89, 139.88, 139.76, 138.39, 137.30, 135.76, 134.66, 132.17, 130.58, 129.89, 128.06, 127.93, 125.65, 125.44, 125.14, 125.10, 124.51, 122.72, 121.01, 120.63, 119.67, 107.50, 69.41, 54.51, 45.34, 37.83, 35.30, 33.63, 31.80, 30.00, 29.33, 29.27, 24.61, 24.42, 22.61, 20.86, 14.06. HRMS-ESI (*m/z*): [M + H]$^+$ Calcd. for ($C_{56}H_{62}N_3O_2S_2$), 872.4283, found: 872.4276.

Synthesis of compound **WS-69**. The **WS-69** was synthesized in a similar manner to that for **WS-67** (115 mg, 65.9%). ^{1}H NMR (400 MHz, THF-d$_8$, *δ*, ppm): 8.12 (s, 1 H), 8.00 (s, 2 H), 7.98 (s, 1 H), 7.86 (s, 1 H), 7.76 (m, 3 H), 7.66 (d, *J* = 7.4 Hz, 1 H), 7.57 (d, *J* = 6.7 Hz, 2 H), 7.14 (d, *J* = 8.0 Hz, 2 H), 7.07 (d, *J* = 8.0 Hz, 2 H), 6.85 (d, *J* = 8.4 Hz, 1 H), 4.83 (m, 1 H), 3.82 (m, 1 H), 2.21 (s, 3 H), 2.00-2.02 (m, 2 H), 1.93-1.96 (m, 4 H), 1.83 (m, 2 H), 1.68-1.72 (m, 1 H) 1.40-1.46 (m, 1 H), 1.07-1.18 (m, 20 H), 0.98 (m, 4 H), 0.68-0.71 (m, 6 H). ^{13}C NMR (100 MHz, THF-d$_8$, *δ*, ppm): 161.27, 158.43, 158.09, 150.63, 147.18, 146.23, 142.22, 138.12, 137.97, 137.42, 136.23, 135.76, 133.77, 129.91, 129.78, 128.63, 127.75, 127.62, 126.14, 125.86, 123.83, 123.72, 123.21, 123.06, 122.43, 120.45, 118.72, 118.57, 118.10, 113.88, 105.21, 100.73, 67.34, 52.52, 43.42, 35.86, 33.31, 31.52, 29.90, 28.10, 27.77, 27.39, 27.36, 20.61, 11.53. HRMS-ESI (m/z): [M + H]$^+$ Calcd. for ($C_{59}H_{63}N_4O_3S_2$), 939.4342, found: 939.4346.

3. DSSCs fabrications

The working electrode composed of the TiO_2 nanoparticle (8 μm nanocrystalline and 8 μm scattering layer) was prepared and modified following the reported procedure.[S3] A screen-printed double layers of TiO_2 particles were used as the photoelectrode (8 μm transparent + 8 μm scattering). A 8 μm thick film of 13 nm TiO_2 particles were first printed on the FTO conducting glass, then kept in a clean box for 10 minutes and dried at 125 °C for 6 min. Afterwards, it was coated by a 8 μm thick second layer of 400 nm light-scattering anatase particles. Finally, the electrodes coated with the TiO_2 pastes were gradually sintered in a muffle furnace at 275 °C for 5 min, at 325 °C for 5 min, at 375 °C for 5 min, at 450 °C for 15 min and at 500 °C for 15 min, respectively. Furthermore, these films were immersed into a 40 mM aqueous $TiCl_4$ solution at 75 °C for 30 min, washed with water and ethanol, and then heated again at 450 °C for 30 min. Then the photoanodes were immersed into a solution of 0.3 mM dyes **WS-66**, **WS-67**, **WS-68** and **WS-69** in a mixture of chloroform and ethanol (volume ratio of 4 : 1) at 25 °C for 12 h without any

coadsorbent. For consensitized DSSCs, a dye solution of 0.3 mM **WS-5** was prepared in a mixture of chloroform and ethanol (volume ratio of 1 : 1). The photoanodes were first dipped in the solution of **WS-68/WS-69** for 12 h, then dipped in the solution of **WS-5** for 2 h at 25 °C, indicated as **WS-68** (12 h) + **WS-5** (2 h) and **WS-69** (12 h) + **WS-5** (2 h), respectively. Then the reversed coadsorption sequence was conducted, the photoanodes were first dipped in **WS-5** solution for 2 h, then dipped in **WS-68/WS-69** solution for 12 h at 25 °C, indicated as **WS-5** (2 h) + **WS-68** (12 h) and **WS-5** (2 h) + **WS-69** (12 h), respectively. The counter electrode was prepared by coating with a drop of H_2PtCl_6 (0.02 M in 2-propanol solution) solution on an FTO plate and heating at 500 °C for 30 min. A hole (diameter of 0.8 mm) was then drilled on the counter electrode. The dye-adsorbed TiO_2 electrode and Pt-counter electrode were sandwiched by a thick hot-melt gasket (25 μm, Surlyn 1702 (DuPont)) and sealed together by pressing them under heat. A drop of electrolyte solution was put on the hole in the back of the counter electrode, and introduced into the cell via vacuum backfilling. Finally, the hole was sealed using a UV-melt gum and a cover glass. The cell area was controlled to be 0.12 cm^2 for photovoltaic performance measurements. The electrolyte used in this work contains 0.6 M of DPMII, 0.05 M of I_2, 0.1 M of LiI, and 0.5 M of TBP in acetonitrile.

4. Measurement equipment and apparatus

^{1}H NMR (400 MHz) and ^{13}C NMR (100 MHz) spectra were obtained by employing a Bruker AM 400 spectrometer. HR-MS measurements were performed using a Waters LCT Premier XE spectrometer. The UV-vis absorption spectra were recorded using a CARY 100 spectrophotometer. Photovoltaic measurements were performed by employing an AM 1.5 solar simulator equipped with a 300 W xenon lamp (model no. 91160, Oriel). The power of the simulated light was calibrated to 100 mW cm^{-2} using a Newport Oriel PV reference cell system (model 91150 V). Photocurrent density-voltage (I-V) curves were obtained by illuminating the cells through the FTO substrate from the photoanode side under standard AM 1.5 conditions with a model 2400 source meter (Keithley Instruments, Inc. USA). The photocurrent action spectra were measured with Newport-74125 system (Newport Instruments). The intensity of the monochromic light was calibrated by a reference silicon cell (Newport-71640). Electrochemical impedance spectroscopy (EIS) for DSSCs was performed using a two-electrode system under dark with electrochemical workstation (Zahner IM6e). The spectra were scanned in a frequency range of 0.1 Hz - 100 kHz under a series of applied bias potential with a magnitude of the alternative signal of 10 mV and characterized using Z-View software.

References

[S1] H. Zhu, Y. Wu, J. Liu, W. Zhang, W. Wu and W. H. Zhu, *J. Mater. Chem. A*, 2015, **3**, 10603.

[S2] M. Zhang, Y. Wang, M. Xu, W. Ma, R. Lia and P. Wang, *Energy Environ. Sci.*, 2013, **6**, 2944.

[S3] Q. Chai, W. Li, Y. Wu, K. Pei, J. Liu, Z. Geng, H. Tian and W. H. Zhu, *ACS Appl. Mater. Interfaces*, 2014, **6**, 14621.

5. Light harvesting efficiency of dyes WS-66, WS-67, WS-68 and WS-69

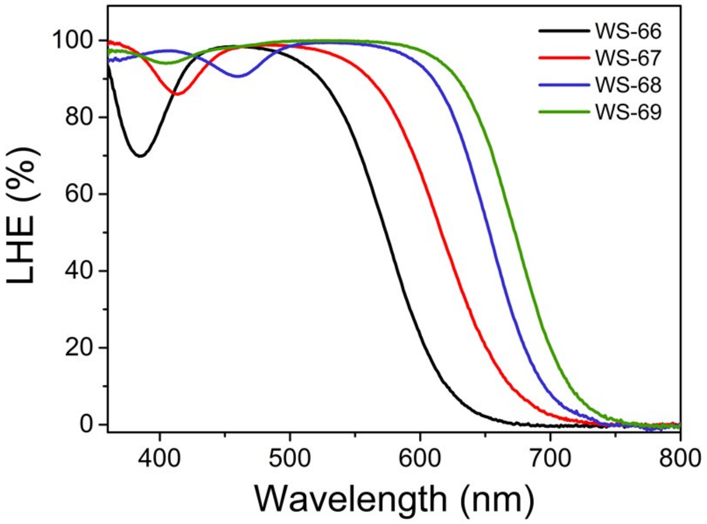

Fig. S1 Light harvesting efficiency of dyes **WS-66**, **WS-67**, **WS-68** and **WS-69** on transparent 8 μm TiO_2 film.

6. Computational details

We employed density functional theory (DFT) calculations to optimize the ground state geometries of the dye sensitizer molecules, using the hybrid B3LYP functional and the 6-31G (d) basis set. At the optimized geometries, time-dependent (TD) DFT calculations were also carried out, using the range-separated CAM-B3LYP functional and the 6-311+G (d, p) basis set. In TDDFT calculations, solvent effects of CH_2Cl_2 were taken into account by the polarizable continuum model. All calculations were carried out using the Gaussian09 program package.

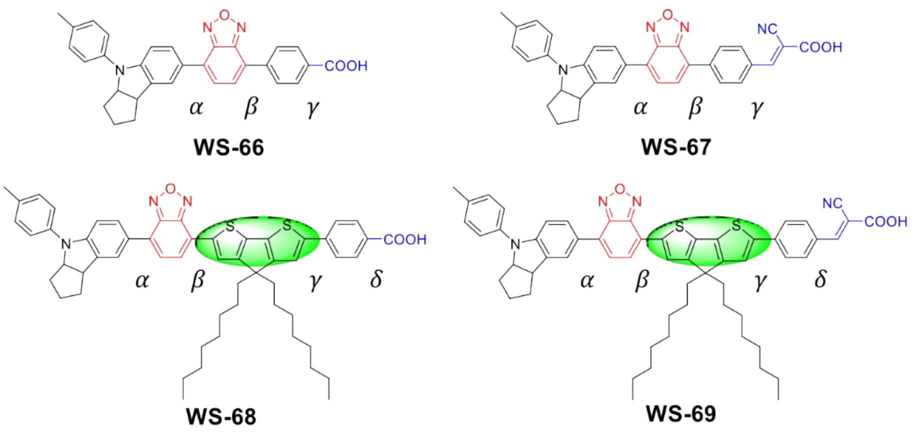

Fig. S2 Molecular structures of dyes **WS-66**, **WS-67**, **WS-68** and **WS-69**.

Table S1 Energy levels (in eV) of frontier molecular orbitals.

Compound	E(HOMO)	E(LUMO)	Gap
**WS-66**	-5.13	-2.50	2.63
**WS-67**	-5.22	-2.86	2.36
**WS-68**	-4.84	-2.55	2.29
**WS-69**	-4.94	-2.83	2.12

Table S2 Computed excitation energy absorption wavelength, oscillator strength and molecular orbital composition for the lowest excited state.

Compound	Excited state	Excitation energy	Oscillator strength	MO composition
**WS-66**	S_1	2.69 eV, 460 nm	0.991	H → L (82%)<br>H-1 → L (13%)
**WS-67**	S_1	2.56 eV, 484 nm	1.367	H → L (69%)<br>H-1 → L (13%)<br>H → L+1 (11%)
**WS-68**	S_1	2.23 eV, 555 nm	1.696	H → L (89%)
	S_2	3.03 eV, 409 nm	0.276	H-1 → L (41%)<br>H → L+1 (38%)
**WS-69**	S_1	2.16 eV, 574 nm	2.302	H → L (72%)<br>H → L+1 (13%)
	S_2	2.71 eV, 457 nm	0.312	H → L+1 (53%)<br>H-1 → L (24%)

Table S3 Dihedral angles between aromatic rings.

Compound	α	β	γ	δ
**WS-66**	25.2°	28.6°	0.1°	–
**WS-67**	25.3°	27.3°	2.2°	–
**WS-68**	25.0°	1.4°	20.4°	0.1°
**WS-69**	24.6°	1.7°	10.6°	0.3°

7. Molecular structures of dye WS-5

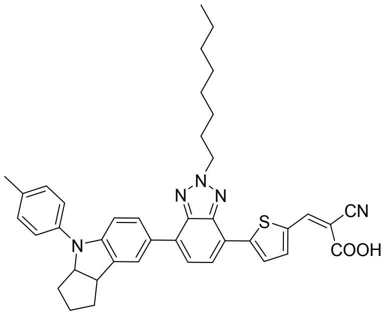

WS-5

Fig. S3 Molecular structures of dye **WS-5**.

8. Optical and electrochemical data for dyes WS-5

Table S4 Optical and electrochemical data for dyes **WS-5** from previous literature.[S3]

Dye	λ_{max} [nm][a]	ε [M^{-1} cm^{-1}][a]	λ_{max} on TiO_2 [nm][b]	HOMO [V][c] (vs NHE)	E_{0-0} [eV][d]	LUMO [V][d] (vs NHE)
**WS-5**	494	19800	446	0.85	2.06	-1.21

9. Adsorption amount of individual dye and cosensitization

Table S5 Adsorption amount of individual dye (**WS-5**, **WS-68** and **WS-69**) and the cosensitization of **WS-68**/**WS-69** with **WS-5** on 16 μm TiO_2 films.

Device	Dye	Adsorption time	Adsorption amount (10^{-7} mol cm^{-2})	percentage in individual dye adsorption	percentage in cosensitization
Individual dye adsorption	**WS-68**	12 h	1.60		-
	**WS-69**	12 h	2.18		-
	**WS-5**	2 h	1.20		-
Cosensitization (**WS-5**+**WS-68**/**WS-69**)	**WS-5**	2 h	0.71	59.17%	47.97%
	**WS-68**	12 h	0.77	48.13%	52.03%
	**WS-5**	2 h	0.11	9.17%	4.89%
	**WS-69**	12 h	2.14	98.17%	95.11%
Cosensitization (**WS-68**/**WS-69**+**WS-5**)	**WS-68**	12 h	0.82	51.25%	48.24%
	**WS-5**	2 h	0.88	73.33%	51.76%
	**WS-69**	12 h	1.87	85.78%	70.83%
	**WS-5**	2 h	0.77	64.17%	29.17%

10. Evolutions of DSSCs based on single WS-69 under visible-light soaking

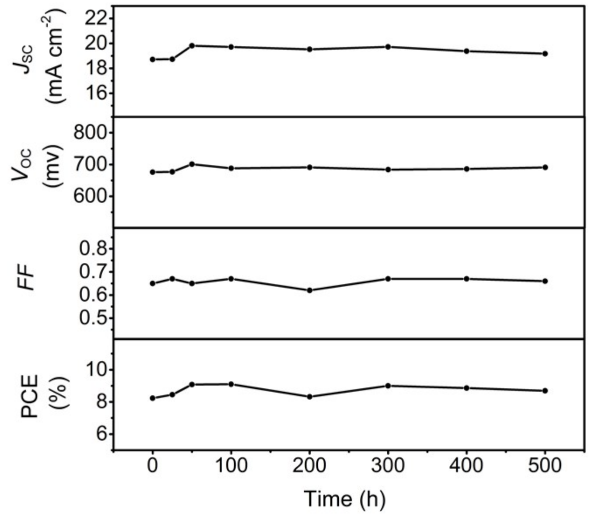

Fig. S4 Evolutions of photovoltaic performance parameters for the DSSCs based on single **WS-69** under visible-light soaking.

11. ^{1}H, ^{13}C NMR and HR-MS of intermediates and tarteted sensitizers

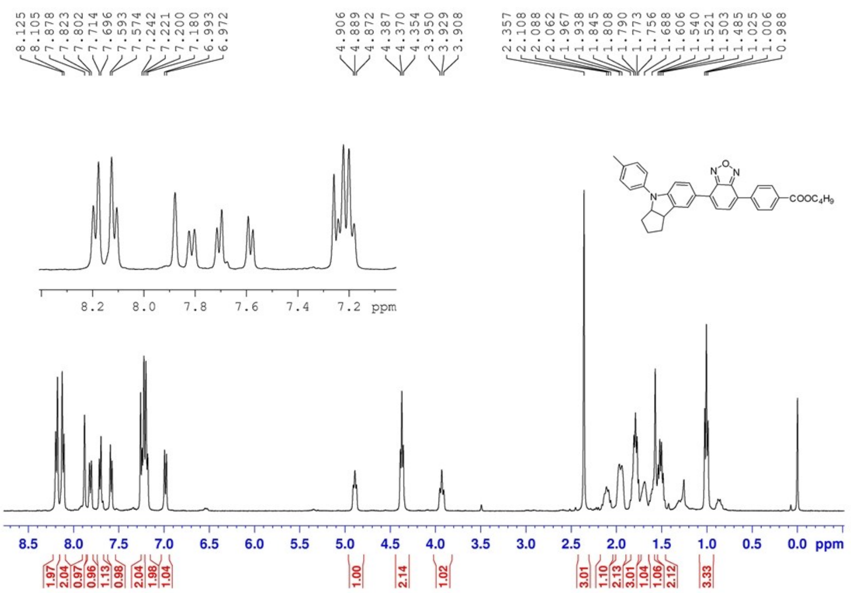

Fig. S5 ^{1}H NMR of compound **3** recorded in $CDCl_3$.

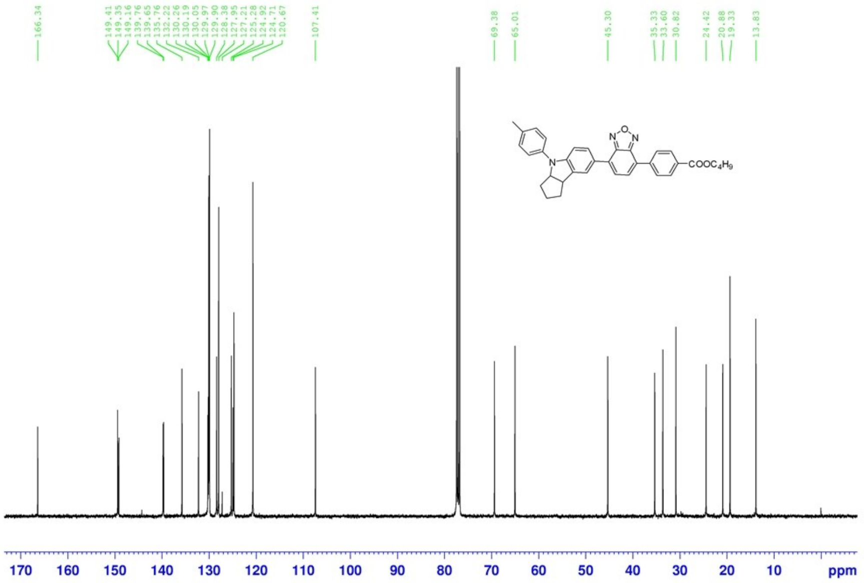

Fig. S6 ^{13}C NMR of compound **3** recorded in $CDCl_3$.

Elemental Composition Report Page 1

Single Mass Analysis
Tolerance = 50.0 PPM / DBE: min = -1.5, max = 100.0
Element prediction: Off
Number of isotope peaks used for i-FIT = 2

Monoisotopic Mass, Even Electron Ions
10 formula(e) evaluated with 1 results within limits (up to 1 closest results for each mass)
Elements Used:
C: 0-35 H: 0-50 N: 0-3 O: 0-3

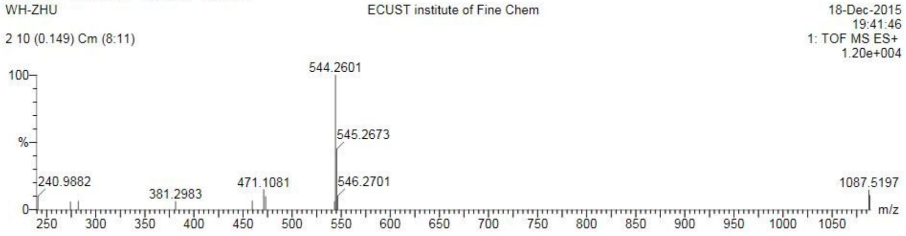

Minimum:				-1.5			
Maximum:		300.0	50.0	100.0			
Mass	Calc. Mass	mDa	PPM	DBE	i-FIT	i-FIT (Norm)	Formula
544.2601	544.2600	0.1	0.2	20.5	15.2	0.0	C35 H34 N3 O3

Fig. S7 High resolution mass spectrometer (HR-MS) of compound **3**.

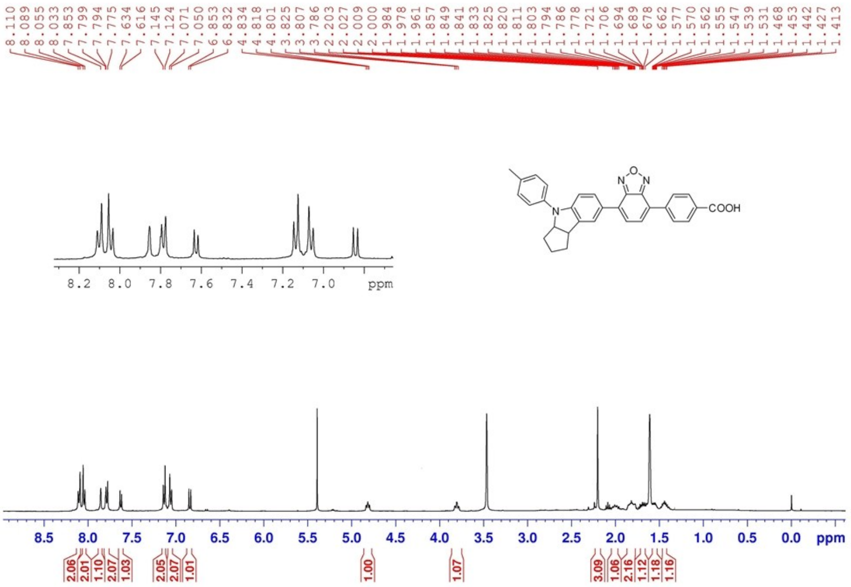

Fig. S8 ^{1}H NMR of **WS-66** recorded in THF-d_8.

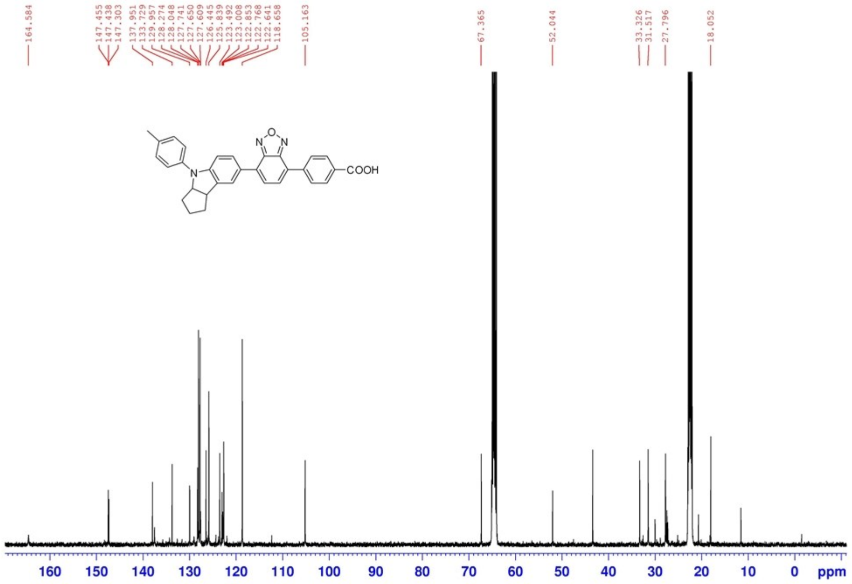

Fig. S9 ^{13}C NMR of **WS-66** recorded in THF-d_8.

Elemental Composition Report **Page 1**

Single Mass Analysis
Tolerance = 50.0 PPM / DBE: min = -1.5, max = 100.0
Element prediction: Off
Number of isotope peaks used for i-FIT = 3

Monoisotopic Mass, Even Electron Ions
56 formula(e) evaluated with 4 results within limits (up to 1 closest results for each mass)
Elements Used:
C: 0-40 H: 0-40 N: 0-3 O: 0-3
WH-ZHU ECUST institute of Fine Chem 17-May-2016 21:04:14
ZW-ZWW-66 6 (0.275) Cm (3:6) 1: TOF MS ES+ 9.05e+003

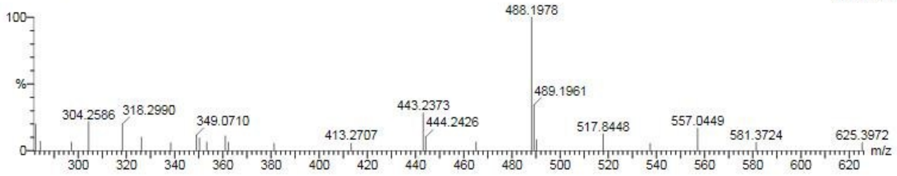

Minimum:				-1.5			
Maximum:		300.0	50.0	100.0			
Mass	Calc. Mass	mDa	PPM	DBE	i-FIT	i-FIT (Norm)	Formula
488.1978	488.1974	0.4	0.8	20.5	16.6	0.0	C31 H26 N3 O3

Fig. S10 High resolution mass spectrometer (HR-MS) of **WS-66**.

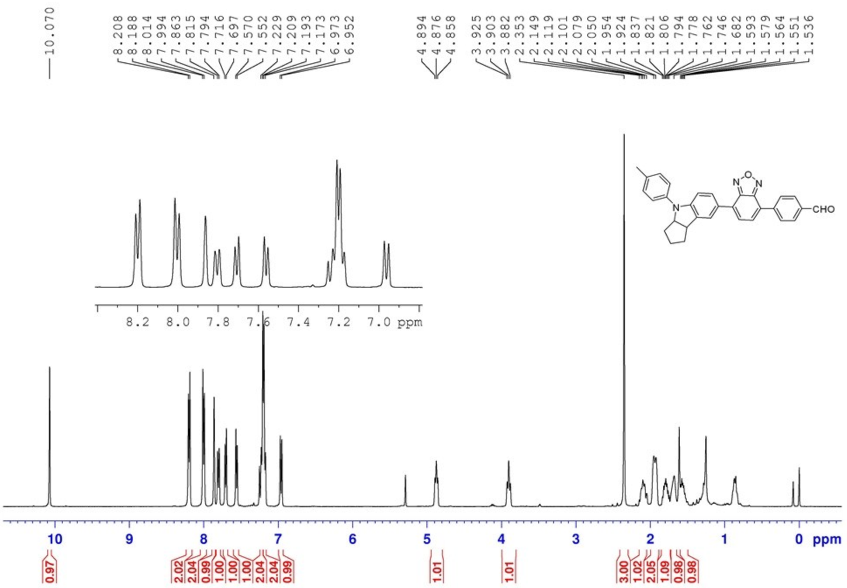

Fig. S11 ^{1}H NMR of compound **5** recorded in $CDCl_3$.

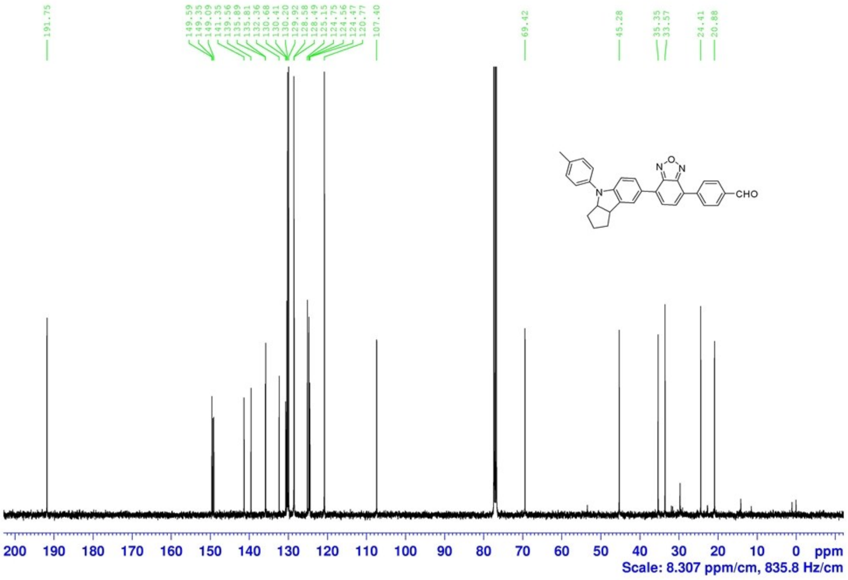

Fig. S12 ^{13}C NMR of compound **5** recorded in $CDCl_3$.

Elemental Composition Report **Page 1**

Single Mass Analysis
Tolerance = 50.0 PPM / DBE: min = -1.5, max = 100.0
Element prediction: Off
Number of isotope peaks used for i-FIT = 2

Monoisotopic Mass, Even Electron Ions
8 formula(e) evaluated with 1 results within limits (up to 1 closest results for each mass)
Elements Used:
C: 0-31 H: 0-50 N: 0-3 O: 0-2
WH-ZHU ECUST institute of Fine Chem 18-Dec-2015 19:32:46
1 18 (0.200) Cm (18:19) 1: TOF MS ES+ 4.21e+003

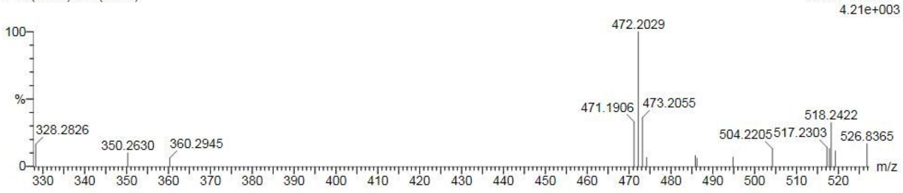

Minimum:				-1.5			
Maximum:		300.0	50.0	100.0			
Mass	Calc. Mass	mDa	PPM	DBE	i-FIT	i-FIT (Norm)	Formula
472.2029	472.2025	0.4	0.8	20.5	7.4	0.0	C31 H26 N3 O2

Fig. S13 High resolution mass spectrometer (HR-MS) of compound **5**.

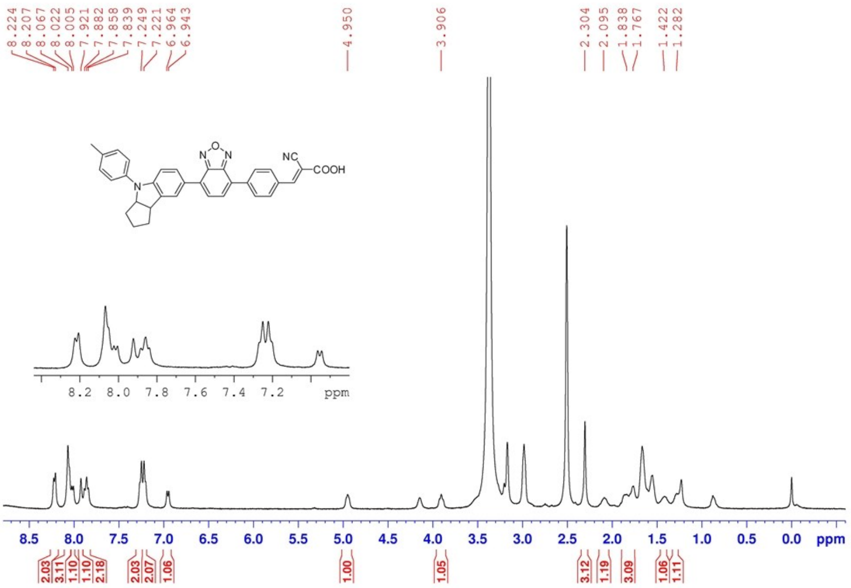

Fig. S14 ^{1}H NMR of **WS-67** recorded in THF-d_8.

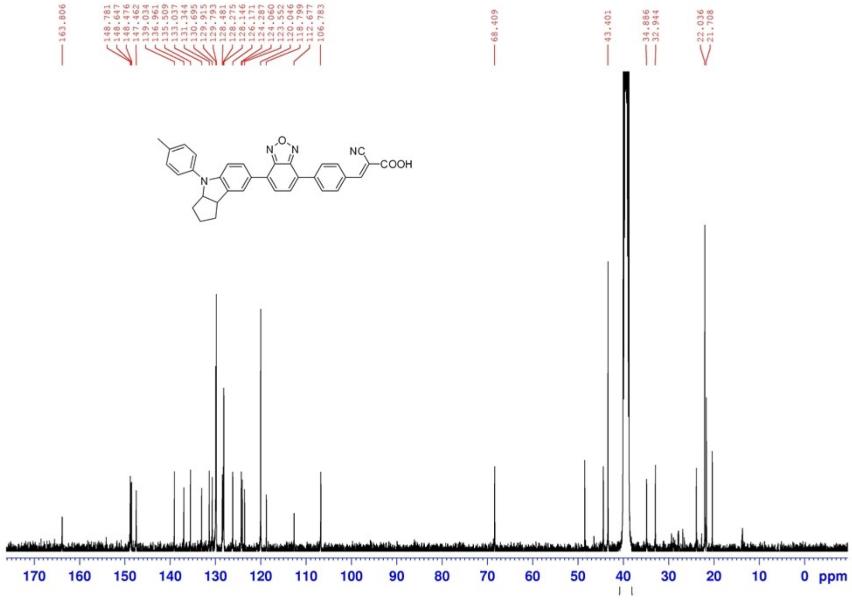

Fig. S15 ^{13}C NMR of **WS-67** recorded in THF-d_8.

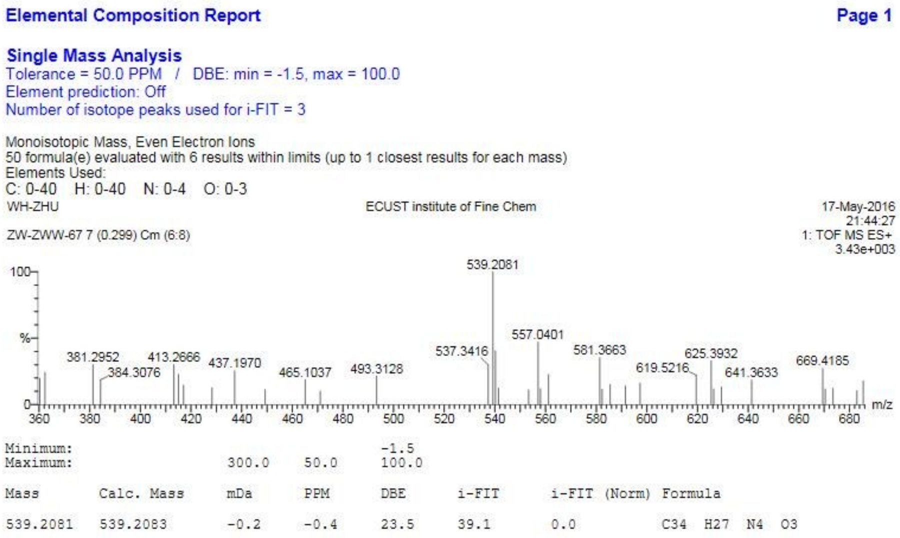

Fig. S16 High resolution mass spectrometer (HR-MS) of **WS-67**.

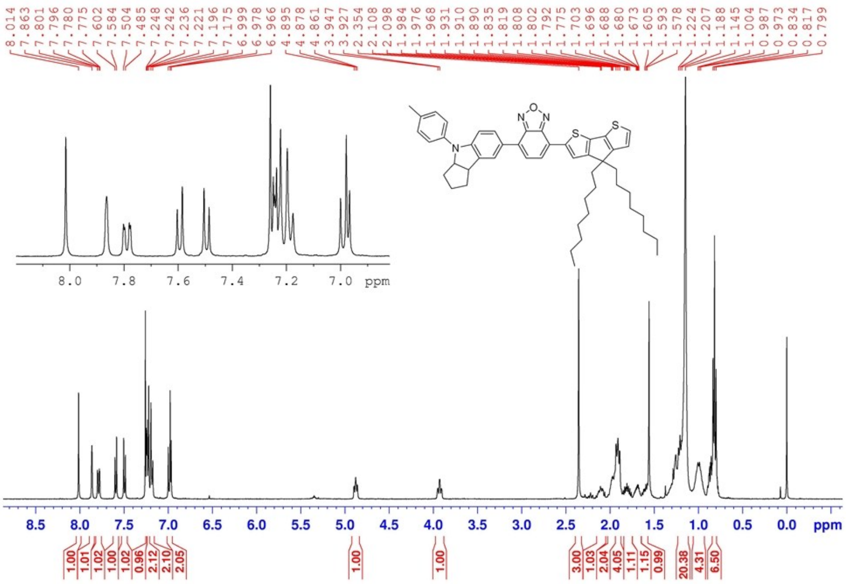

Fig. S17 ^{1}H NMR of compound **7** recorded in $CDCl_3$.

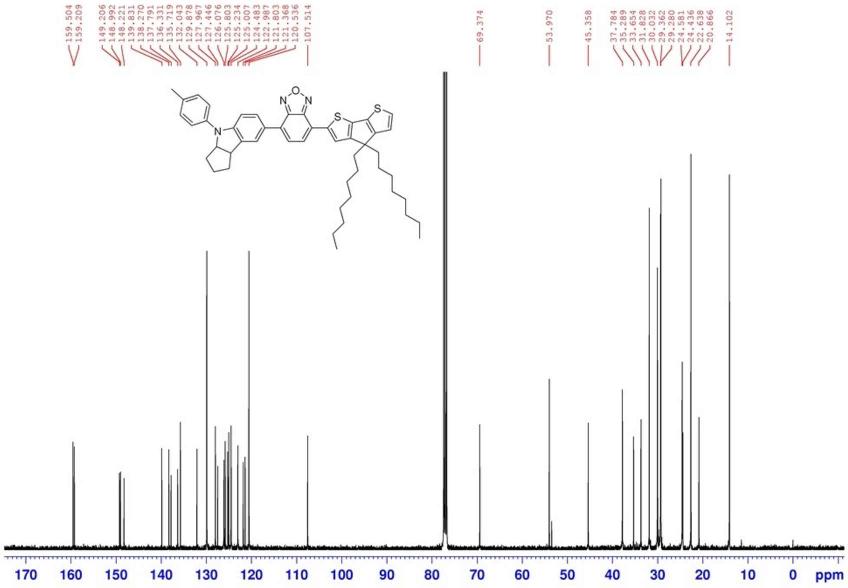

Fig. S18 ^{13}C NMR of compound **7** recorded in $CDCl_3$.

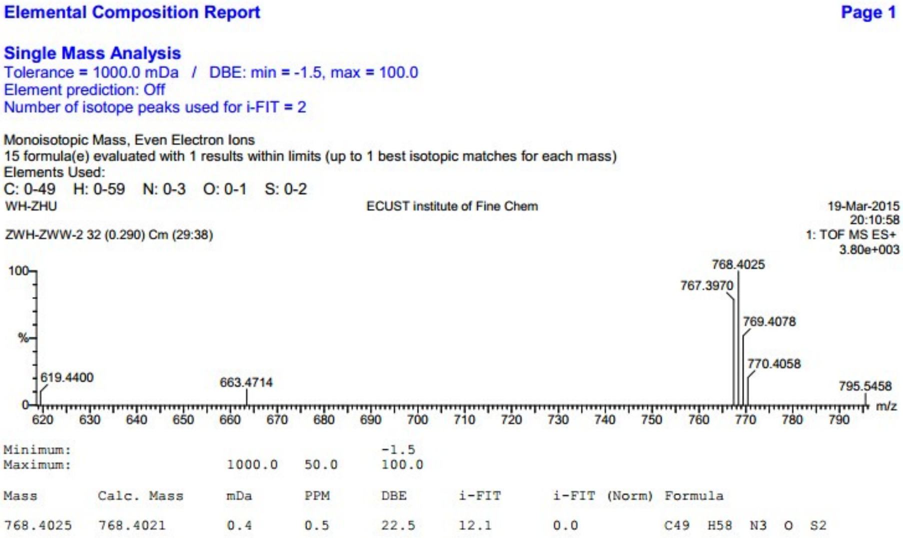

Fig. S19 High resolution mass spectrometer (HR-MS) of compound **7**.

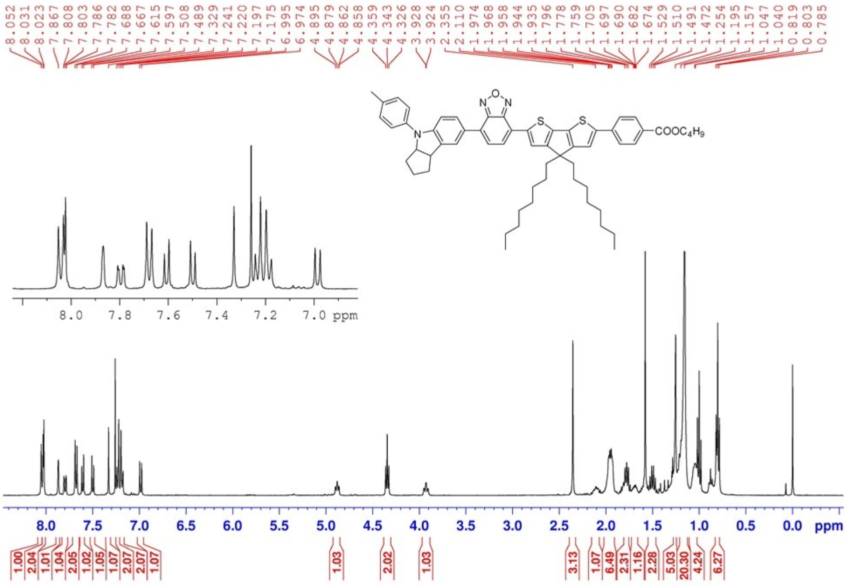

Fig. S20 ^{1}H NMR of compound **9** recorded in $CDCl_3$.

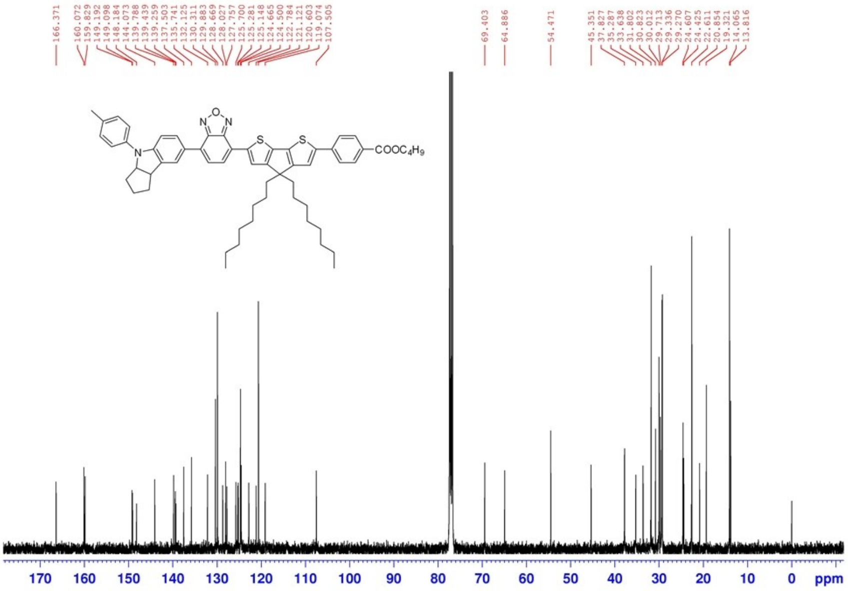

Fig. S21 ^{13}C NMR of compound **9** recorded in $CDCl_3$.

Elemental Composition Report Page 1

Single Mass Analysis
Tolerance = 50.0 PPM / DBE: min = -1.5, max = 100.0
Element prediction: Off
Number of isotope peaks used for i-FIT = 2

Monoisotopic Mass, Even Electron Ions
37 formula(e) evaluated with 1 results within limits (up to 1 closest results for each mass)
Elements Used:
C: 0-60 H: 0-100 N: 0-3 O: 0-3 S: 0-2

WH-ZHU ECUST institute of Fine Chem 23-Oct-2015 21:55:14

ZWH-ZWW-1 107 (0.751) Cm (107:121) 1: TOF MS ES+ 3.11e+004

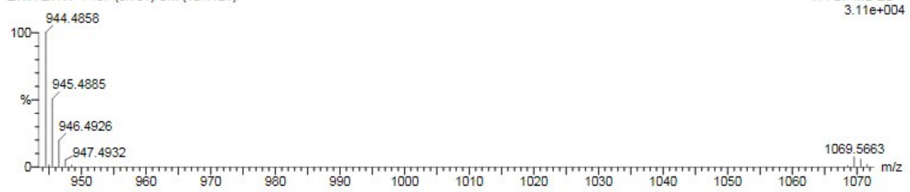

Mass	Calc. Mass	mDa	PPM	DBE	i-FIT	i-FIT (Norm)	Formula
Minimum:				-1.5			
Maximum:		300.0	50.0	100.0			
944.4858	944.4859	-0.1	-0.1	27.5	30.7	0.0	C60 H70 N3 O3 S2

Fig. S22 High resolution mass spectrometer (HR-MS) of compound **9**.

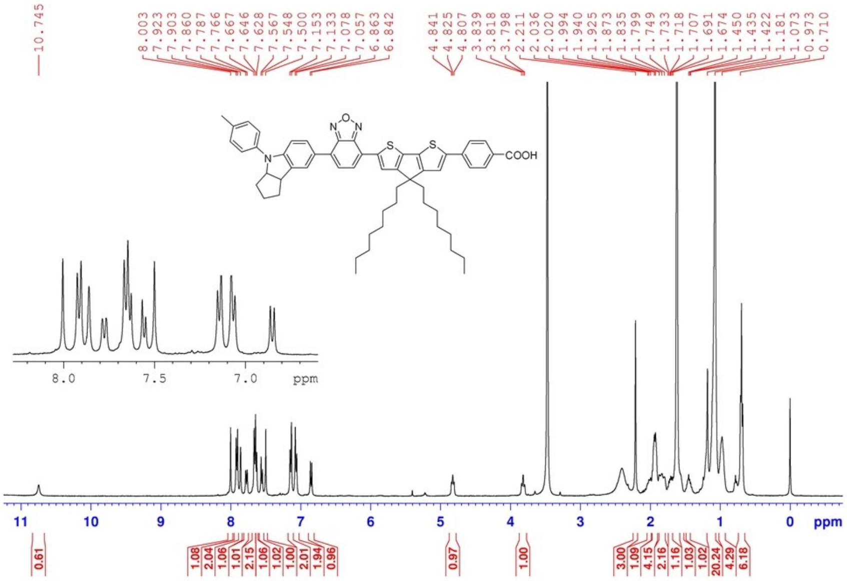

Fig. S23 ^{1}H NMR of **WS-68** recorded in THF-d_8.

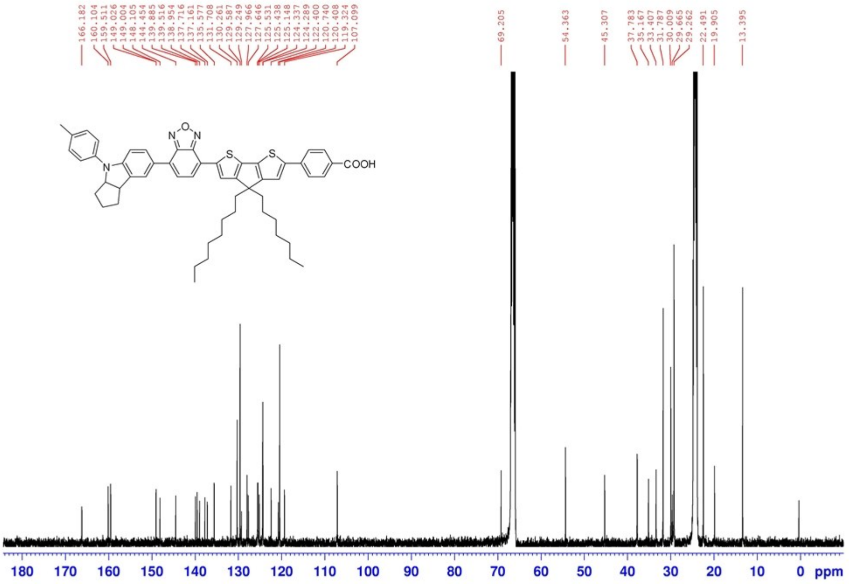

Fig. S24 ^{13}C NMR of **WS-68** recorded in THF-d_8.

Elemental Composition Report Page 1

Single Mass Analysis
Tolerance = 50.0 PPM / DBE: min = -1.5, max = 100.0
Element prediction: Off
Number of isotope peaks used for i-FIT = 3

Monoisotopic Mass, Even Electron Ions
29 formula(e) evaluated with 1 results within limits (up to 1 closest results for each mass)
Elements Used:
C: 0-56 H: 0-62 N: 0-3 O: 0-3 S: 0-2

WH-ZHU ECUST institute of Fine Chem 13-Jul-2016 20:51:55

ZW-ZWW-1 46 (0.371) Cm (42:48) 1: TOF MS ES+ 1.18e+004

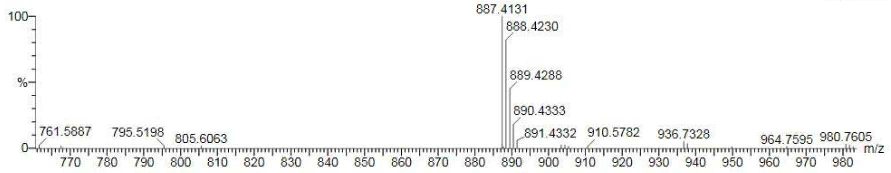

Minimum:				-1.5			
Maximum:		300.0	50.0	100.0			
Mass	Calc. Mass	mDa	PPM	DBE	i-FIT	i-FIT (Norm)	Formula
888.4230	888.4233	-0.3	-0.3	27.5	22.1	0.0	C56 H62 N3 O3 S2

Fig. S25 High resolution mass spectrometer (HR-MS) of **WS-68**.

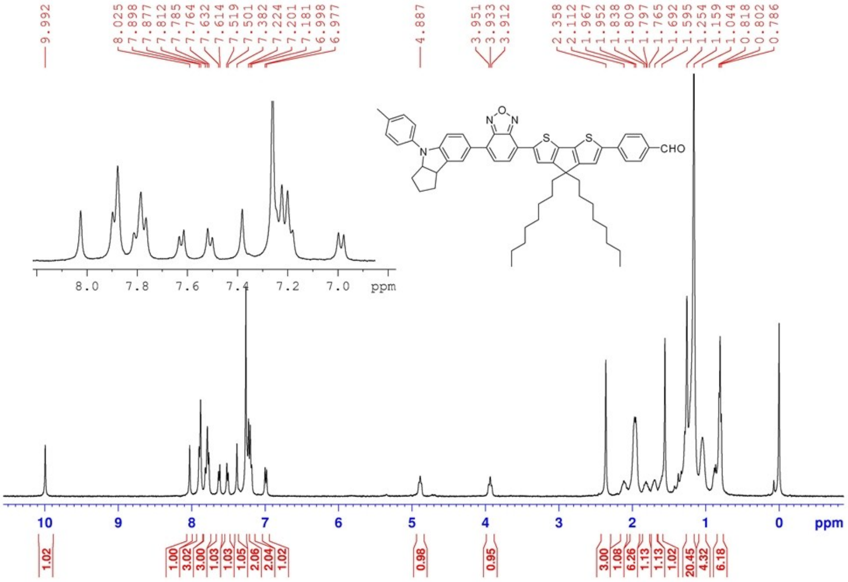

Fig. S26 ^{1}H NMR of compound **10** recorded in $CDCl_3$.

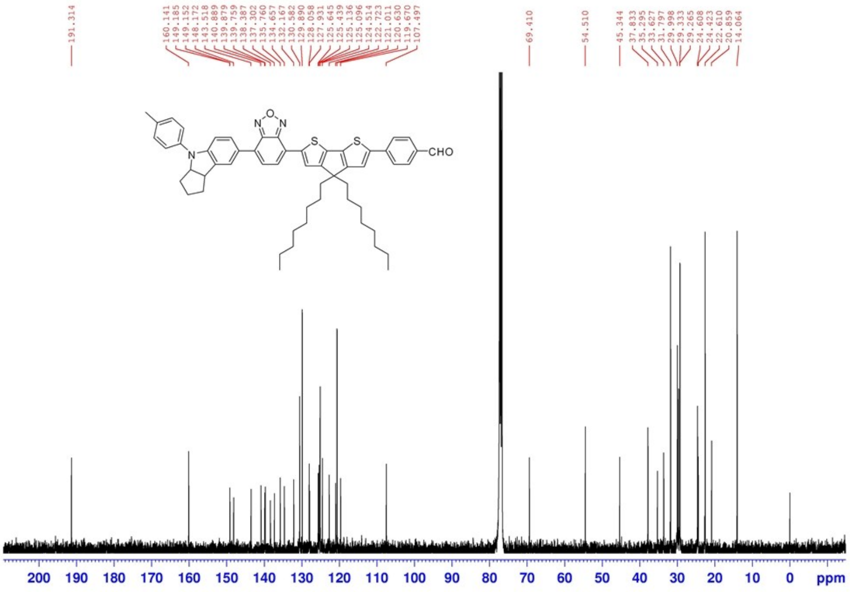

Fig. S27 ^{13}C NMR of compound **10** recorded in $CDCl_3$.

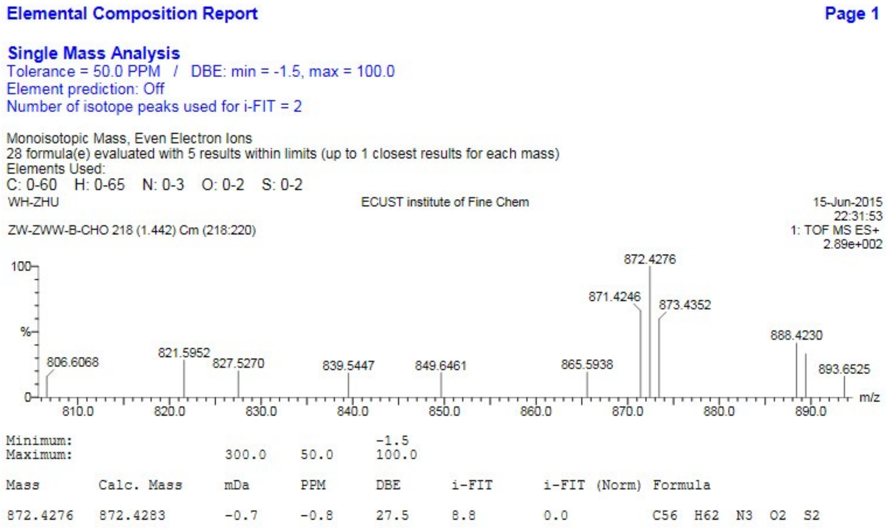

Fig. S28 High resolution mass spectrometer (HR-MS) of compound **10**.

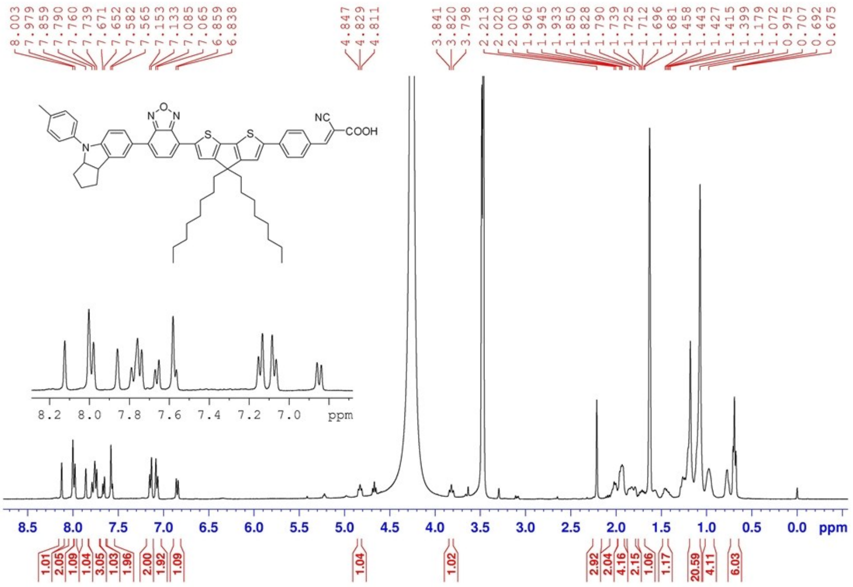

Fig. S29 ^{1}H NMR of **WS-69** recorded in THF-d_8.

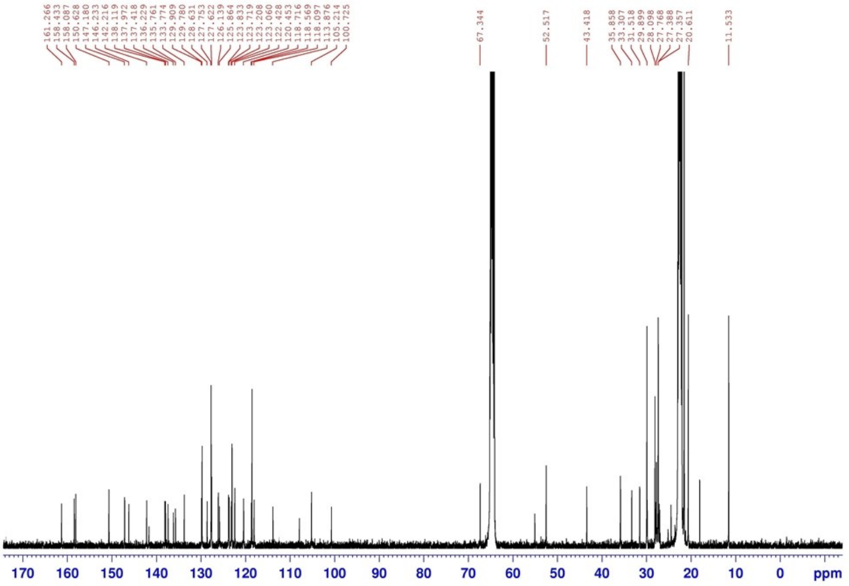

Fig. S30 ^{13}C NMR of **WS-69** recorded in THF-d_8.

Elemental Composition Report **Page 1**

Single Mass Analysis
Tolerance = 50.0 PPM / DBE: min = -1.5, max = 100.0
Element prediction: Off
Number of isotope peaks used for i-FIT = 2

Monoisotopic Mass, Even Electron Ions
33 formula(e) evaluated with 1 results within limits (up to 1 closest results for each mass)
Elements Used:
C: 0-60 H: 0-65 N: 0-4 O: 0-3 S: 0-2

WH-ZHU ECUST institute of Fine Chem 15-Jun-2015 22:37:33
ZW-ZWW-B-ZHONG 26 (0.249) Cm (24:28) 1: TOF MS ES+ 1.53e+003

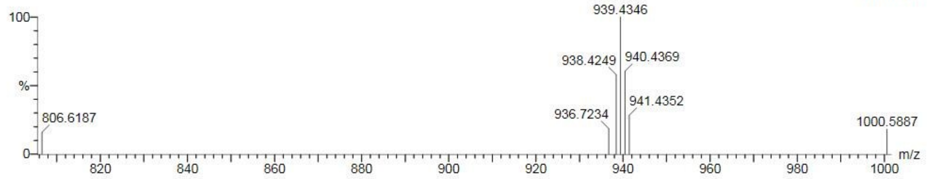

Minimum: -1.5
Maximum: 300.0 50.0 100.0

Mass	Calc. Mass	mDa	PPM	DBE	i-FIT	i-FIT (Norm)	Formula
939.4346	939.4342	0.4	0.4	30.5	10.9	0.0	C59 H63 N4 O3 S2

Fig. S31 High resolution mass spectrometer (HR-MS) of **WS-69**.
